# Management of intra-abdominal infections: recommendations by the Italian council for the optimization of antimicrobial use

**DOI:** 10.1186/s13017-024-00551-w

**Published:** 2024-06-08

**Authors:** Massimo Sartelli, Carlo Tascini, Federico Coccolini, Fabiana Dellai, Luca Ansaloni, Massimo Antonelli, Michele Bartoletti, Matteo Bassetti, Federico Boncagni, Massimo Carlini, Anna Maria Cattelan, Arturo Cavaliere, Marco Ceresoli, Alessandro Cipriano, Andrea Cortegiani, Francesco Cortese, Francesco Cristini, Eugenio Cucinotta, Lidia Dalfino, Gennaro De Pascale, Francesco Giuseppe De Rosa, Marco Falcone, Francesco Forfori, Paola Fugazzola, Milo Gatti, Ivan Gentile, Lorenzo Ghiadoni, Maddalena Giannella, Antonino Giarratano, Alessio Giordano, Massimo Girardis, Claudio Mastroianni, Gianpaola Monti, Giulia Montori, Miriam Palmieri, Marcello Pani, Ciro Paolillo, Dario Parini, Giustino Parruti, Daniela Pasero, Federico Pea, Maddalena Peghin, Nicola Petrosillo, Mauro Podda, Caterina Rizzo, Gian Maria Rossolini, Alessandro Russo, Loredana Scoccia, Gabriele Sganga, Liana Signorini, Stefania Stefani, Mario Tumbarello, Fabio Tumietto, Massimo Valentino, Mario Venditti, Bruno Viaggi, Francesca Vivaldi, Claudia Zaghi, Francesco M. Labricciosa, Fikri Abu-Zidan, Fausto Catena, Pierluigi Viale

**Affiliations:** 1 Department of Surgery, Macerata Hospital, Via Santa Lucia 2, 62100 Macerata, Italy; 2 Infectious Diseases Clinic, Santa Maria Della Misericordia University Hospital of Udine, ASUFC, Udine, Italy; 3 Infectious Diseases Clinic, Department of Medicine (DAME), University of Udine, Udine, Italy; 4 Department of General, Emergency and Trauma Surgery, Azienda Ospedaliero Universitaria Pisana, University Hospital, Pisa, Italy; 5 Division of General Surgery, Fondazione IRCCS Policlinico San Matteo, Pavia, Italy; 6 Department of Clinical, Diagnostic and Pediatric Sciences, University of Pavia, Pavia, Italy; 7 Dipartimento di Scienze Biotecnologiche di Base, Cliniche Intensivologiche e Perioperatorie, Università Cattolica del Sacro Cuore, Rome, Italy; 8 Dipartimento di Scienze Dell’Emergenza, Anestesiologiche e Della Rianimazione, Fondazione Policlinico Universitario A. Gemelli IRCCS, Rome, Italy; 9 Department of Biomedical Sciences, Humanitas University, Milan, Italy; 10 Infectious Disease Unit, IRCCS Humanitas Research Hospital, Milan, Italy; 11 Division of Infectious Diseases, Department of Health Sciences, University of Genova, Genoa, Italy; 12 Division of Infectious Diseases, IRCCS Ospedale Policlinico San Martino, Genoa, Italy; 13 Anesthesiology and Intensive Care Unit, Macerata Hospital, Macerata, Italy; 14 Department of General Surgery, S. Eugenio Hospital, Rome, Italy; 15 Infectious and Tropical Diseases Unit, Padua University Hospital, Padua, Italy; 16 Department of Molecular Medicine, University of Padua, Padua, Italy; 17 Unit of Hospital Pharmacy, Viterbo Local Health Authority, Viterbo, Italy; 18 General and Emergency Surgery, Milano-Bicocca University, School of Medicine and Surgery, Monza, Italy; 19 Department of Emergency Medicine, Azienda Ospedaliero Universitaria Pisana, University of Pisa, Pisa, Italy; 20 Department of Precision Medicine in Medical Surgical and Critical Care, University of Palermo, Palermo, Italy; 21 Department of Anesthesia, Intensive Care and Emergency, Policlinico Paolo Giaccone, University of Palermo, Palermo, Italy; 22 Emergency Surgery Unit, San Filippo Neri Hospital, Rome, Italy; 23 Infectious Diseases Unit, AUSL Romagna, Forlì and Cesena Hospitals, Forlì, Italy; 24 Department of Medical and Surgical Sciences, Alma Mater Studiorum University of Bologna, Bologna, Italy; 25 Department of Human Pathology of the Adult and Evolutive Age “Gaetano Barresi”, Section of General Surgery, University of Messina, Messina, Italy; 26 Anesthesia and Intensive Care Unit, Department of Precision and Regenerative Medicine and Ionian Area, Polyclinic of Bari, University of Bari, Bari, Italy; 27 Department of Medical Sciences, Infectious Diseases, University of Turin, Turin, Italy; 28 Infectious Diseases Unit, Department of Clinical and Experimental Medicine, Azienda Ospedaliero Universitaria Pisana, University of Pisa, Pisa, Italy; 29 Anesthesia and Intensive Care, Anesthesia and Resuscitation Department, Azienda Ospedaliero Universitaria Pisana, University of Pisa, Pisa, Italy; 30 Department of Medical and Surgical Sciences, Alma Mater Studiorum University of Bologna, Bologna, Italy; 31 Clinical Pharmacology Unit, Department for Integrated Infectious Risk Management, IRCCS Azienda Ospedaliero-Universitaria Di Bologna, Bologna, Italy; 32 Section of Infectious Diseases, Department of Clinical Medicine and Surgery, University of Naples Federico II, Naples, Italy; 33 Department on Clinical and Experimental Medicine, University of Pisa, Pisa, Italy; 34 Infectious Diseases Unit, Department for Integrated Infectious Risk Management, IRCCS Azienda Ospedaliero-Universitaria di Bologna, Bologna, Italy; 35 Unit of Emergency Surgery, Careggi University Hospital, Florence, Italy; 36 Anesthesia and Intensive Care Medicine, Policlinico di Modena, University of Modena and Reggio Emilia, Modena, Italy; 37 Department of Public Health and Infectious Diseases, AOU Policlinico Umberto 1, Sapienza University of Rome, Rome, Italy; 38 Department of Anesthesia and Intensive Care, ASST GOM Niguarda Ca’ Granda, Milan, Italy; 39 Unit of General and Emergency Surgery, Vittorio Veneto Hospital, Vittorio Veneto, Italy; 40 Hospital Pharmacy Unit, Fondazione Policlinico Universitario A. Gemelli IRCCS, Rome, Italy; 41 Emergency Department, University of Verona, Verona, Italy; 42 General Surgery Department, Santa Maria Della Misericordia Hospital, Rovigo, Italy; 43 Infectious Diseases Unit, Pescara General Hospital, Pescara, Italy; 44 Department of Emergency, Anaesthesia and Intensive Care Unit, ASL1 Sassari, Sassari, Italy; 45 Department of Medicine, Surgery and Pharmacy, University of Sassari, Sassari, Italy; 46 Infectious and Tropical Diseases Unit, Department of Medicine and Surgery, University of Insubria-ASST-Sette Laghi, Varese, Italy; 47 Infection Prevention and Control Service, Fondazione Policlinico Universitario Campus Bio-Medico, Rome, Italy; 48 Department of Surgical Science, University of Cagliari, Cagliari, Italy; 49 Department of Translational Research and New Technologies in Medicine and Surgery, University of Pisa, Pisa, Italy; 50 Department of Experimental and Clinical Medicine, University of Florence, Florence, Italy; 51 Microbiology and Virology Unit, Florence Careggi University Hospital, Florence, Italy; 52 Department of Medical and Surgical Sciences, “Magna Graecia” University, Catanzaro, Italy; 53 Infectious and Tropical Disease Unit, “Renato Dulbecco” Teaching Hospital, Catanzaro, Italy; 54 Hospital Pharmacy Unit, Macerata Hospital, AST Macerata, Macerata, Italy; 55 Emergency and Trauma Surgery Unit, Fondazione Policlinico Universitario A Gemelli IRCCS, Rome, Italy; 56 Department of Medical and Surgical Sciences, Università Cattolica del Sacro Cuore, Rome, Italy; 57 Unit of Infectious and Tropical Diseases, ASST Spedali Civili Di Brescia, Brescia, Italy; 58 Department of Biomedical and Biotechnological Sciences (BIOMETEC), University of Catania, Catania, Italy; 59 Department of Medical Biotechnologies, University of Siena, Siena, Italy; 60 Infectious and Tropical Diseases Unit, Azienda Ospedaliero-Universitaria Senese, Siena, Italy; 61 UO Antimicrobial Stewardship-AUSL Bologna, Bologna, Italy; 62 Radiology Department, Sant’Antonio Abate Hospital, Tolmezzo, Italy; 63 Department of Public Health and Infectious Diseases, Sapienza University of Rome, Rome, Italy; 64 Intensive Care Department, Careggi Hospital, Florence, Italy; 65 Hospital Pharmacy Unit, Asl Toscana Nord Ovest, Pisa, Italy; 66 General, Emergency and Trauma Surgery Department, Vicenza Hospital, Vicenza, Italy; 67 Global Alliance for Infections in Surgery, Macerata, Italy; 68 Statistics and Research Methodology, The Research Office, College of Medicine and Health Sciences, United Arab Emirates University, Al-Ain, United Arab Emirates; 69 Emergency and General Surgery Department, Bufalini Hospital, Cesena, Italy

**Keywords:** Antimicrobial resistance, Antimicrobial therapy, Intra-abdominal infections, Source control

## Abstract

Intra-abdominal infections (IAIs) are common surgical emergencies and are an important cause of morbidity and mortality in hospital settings, particularly if poorly managed. The cornerstones of effective IAIs management include early diagnosis, adequate source control, appropriate antimicrobial therapy, and early physiologic stabilization using intravenous fluids and vasopressor agents in critically ill patients. Adequate empiric antimicrobial therapy in patients with IAIs is of paramount importance because inappropriate antimicrobial therapy is associated with poor outcomes. Optimizing antimicrobial prescriptions improves treatment effectiveness, increases patients’ safety, and minimizes the risk of opportunistic infections (such as *Clostridioides difficile*) and antimicrobial resistance selection. The growing emergence of multi-drug resistant organisms has caused an impending crisis with alarming implications, especially regarding Gram-negative bacteria. The Multidisciplinary and Intersociety Italian Council for the Optimization of Antimicrobial Use promoted a consensus conference on the antimicrobial management of IAIs, including emergency medicine specialists, radiologists, surgeons, intensivists, infectious disease specialists, clinical pharmacologists, hospital pharmacists, microbiologists and public health specialists. Relevant clinical questions were constructed by the Organizational Committee in order to investigate the topic. The expert panel produced recommendation statements based on the best scientific evidence from PubMed and EMBASE Library and experts’ opinions. The statements were planned and graded according to the Grading of Recommendations Assessment, Development and Evaluation (GRADE) hierarchy of evidence. On November 10, 2023, the experts met in Mestre (Italy) to debate the statements. After the approval of the statements, the expert panel met via email and virtual meetings to prepare and revise the definitive document. This document represents the executive summary of the consensus conference and comprises three sections. The first section focuses on the general principles of diagnosis and treatment of IAIs. The second section provides twenty-three evidence-based recommendations for the antimicrobial therapy of IAIs. The third section presents eight clinical diagnostic-therapeutic pathways for the most common IAIs. The document has been endorsed by the Italian Society of Surgery.

## Background

Intra-abdominal infections (IAIs) are common surgical emergencies and represent an important intra-hospital cause of morbidity and mortality, especially if poorly treated. IAIs represent a notable factor contributing to the loss of both human lives and resources across global hospital settings. The WISS study [1] reported an estimated overall mortality rate of 9.2% among patients affected by complicated intra-abdominal infections (cIAIs) globally. The grading of the clinical severity of patients with cIAIs has been well described by the sepsis definitions. The data from WISS study showed that mortality was significantly affected by sepsis status when divided into four categories. Mortality rates increase in patients developing organ dysfunction and septic shock. Mortality by sepsis status was as follows: no sepsis 1.2%, sepsis only 4.4%, severe sepsis 27.8%, and septic shock 67.8%.

Despite still high mortality, short-term survival from sepsis of abdominal origin has improved in recent years [2, 3]. As a result, there is a growing population of sepsis survivors, and with rapid implementation of evidence-based care, early mortality has decreased substantially, but many sepsis survivors are now progressing into chronic critical illness with poorly defined long-term outcomes [4]. These patients frequently experience new symptoms, long-term disability [5], worsening of chronic health conditions, and increased risk for death following sepsis hospitalization [6].

Defining the patient with IAIs at high risk for failure is difficult. “High risk” may be attributed to the patient’s underlying condition(s), such as age, comorbidity or the disease severity status on presentation. However, a “low-risk” patient may be converted to “high risk” if the care provider loses the “window of opportunity” to diagnose, resuscitate, and start timely treatment. Thus, there are numerous situations that must be taken into account when addressing high-risk patients and treatment failure.

In general, high-risk IAI is attributed to patient factors (advanced age, immunosuppression, malignant disease, and pre-existing medical comorbidities) or disease factors, represented by high-risk scores (such as ASA, APACHE, SOFA scores), delay in intervention (usually > 24 h), inability to obtain source control, and an IAI that is hospital acquired (rather than community acquired).

The cornerstones of IAIs management encompass timely diagnosis, adequate source control, early and appropriate antimicrobial therapy, and expeditious physiological stabilization through intravenous fluid therapy and vasopressor agents, in critically ill patients.

## Principles of intra-abdominal infections management

### Diagnosis

In patients with suspected IAIs, a step-up approach for clinical, instrumental, and laboratory diagnosis should be proposed according to the patients’ clinical conditions.

Diagnosis of IAIs is based primarily on clinical assessment. Typically, the patient is admitted to the emergency department with abdominal pain that may be associated with signs of systemic inflammatory response syndrome, including fever, tachycardia, tachypnoea and leucocytosis or leukopenia. Abdominal rigidity suggests the presence of a cIAI, while hypotension, polypnea or dyspnoea and acute altered mental status may warn signs of the patient’s transition to sepsis. Unfortunately, physical evaluation sometimes can be compromised by a variety of clinical constraints like impaired consciousness or severe underlying disease.

White blood count is one of the most common laboratory exams performed when a cIAI is suspected. However, leucocytosis is poorly sensitive and relatively non-specific in these infections. C-reactive protein (CRP) is an acute-phase protein rapidly released during inflammation, increasing especially during bacterial infections because of the induction of an intense inflammatory reaction. It serves as an indirect marker of both inflammation and infection. Conversely, bacterial infections can cause a rapid increase in procalcitonin (PCT), while viral infections or non-infectious inflammation hardly can affect it [7, 8]. Several conditions, including trauma, acute or chronic renal impairment and recent surgery can influence both these markers. Therefore, it is important to include PCT and CRP levels in clinical algorithms and interpret them in the clinical context. An increase in serum lactate is typically observed in conditions where there is an imbalance between oxygen demand and its supply. Anaerobic metabolism can result in a reduction in arterial oxygen content (hypoxemia), decreased perfusion pressure (hypotension), misdistribution of flow and diminished oxygen diffusion across capillary membranes. Hyperlactatemia is associated with an augmented risk of mortality [9]. The lactate level has been proposed as a useful screening marker for suspected sepsis in adult patients [10, 11].

Ultrasonography (US) and computed tomography (CT) scan are essential diagnostic tools in managing IAIs. US is the most suitable imaging modality for critically ill unstable patients who cannot be moved from the intensive care unit (ICU) for additional imaging. US can be performed at the bedside and easily repeated, even though it depends on the operator's skills. Ileus and obesity can limit its value by restricting the sonographic window. US is the preferred initial diagnostic investigation for acute cholecystitis or thoracic fluid collections.

In all the other conditions (stable patients with non-biliary IAIs), a contrast-enhanced triple phase CT scan is the gold standard in diagnosing and staging IAIs patients. It provides a standardised and operator-independent evaluation and can provide a multisectoral body regions evaluation that may facilitate the identification of the source of infection within a few minutes. Contrast-enhanced CT scan offers improved anatomical details of the intestinal wall, allowing for the detection of both primary and secondary pathologies in the surrounding mesentery. Additionally, it can highlight segmental intestinal ischemia and extraluminal air in the peritoneal cavity [12]. It also provides information about the treatment strategy, guiding clinicians in defining the adequate management pathway for each patient. Intravenous contrast medium is sometimes withheld because of the risk of complication in patients undergoing CT scan for abdominal pain. In a multicenter retrospective diagnostic accuracy study of 201 consecutive adult emergency department (ED) patients who underwent a CT scan, unenhanced CT was approximately 30% less accurate than contrast-enhanced CT for evaluating abdominal pain in the ED. In many patients, the risk of withholding iodinated contrast medium may be higher than the risk of administering it [13]. In 2019, a Cochrane systematic review assessed the accuracy of CT scans in diagnosing acute appendicitis in adults [14]. Overall sensitivity was 0.95, and specificity was 0.94. In subgroup analyses the sensitivity was higher in the intravenous contrast-enhanced CT scan (0.96, 95% confidence interval [CI] 0.92–0.98), rectal contrast-enhanced CT scan (0.97, 95% CI 0.93–0.99), and both intravenous- and oral-enhanced CT scan (0.96, 95% CI 0.93–0.98) compared to non-enhanced CT scan (0.91, 95% CI 0.87–0.93). Although it represents the most sensitive imaging investigation for patients with IAIs, a step-up approach with CT performed after an inconclusive or negative US has been proposed as a safe alternative approach for patients with IAIs, especially in the setting of acute diverticulitis and acute appendicitis [15, 16].

According to the current literature, magnetic resonance imaging (MRI) may play a significant role in cIAIs [17]. However, the challenges of performing MRI in emergency settings limit its routine application. MRI provides at least the same sensitivity and specificity as CT, and despite higher costs and potential availability issues in many centres, it should be considered a first-line imaging examination for pregnant women [17].

### Source control

Source control is of paramount importance in managing patients with IAIs. The term source control encompasses all those physical procedures used to control a focus of infection and to restore the optimal function of the affected area [18].

Effective source control requires a deep understanding of pathophysiology, complexities of the infection responses, surgical and non-surgical options and often requires the definition of an adequate balance between therapeutic aggressiveness and judicious decision-making. An adequate source control intervention can rapidly improve the course of IAIs. However, an improper decision may change a difficult clinical challenge into a clinical burden. Adequate source control can also reduce antibiotic use, allowing short courses of antibiotic therapy. Both operative and non-operative techniques can achieve control of the source of infection. Surgery remains the most viable therapeutic strategy for managing surgical infections in critically ill patients. The decision for a specific source control procedure should be defined according to patients’ and infection’s characteristics, as well as the availability of technical expertise at the local institution.

Non-operative interventional procedures include percutaneous US- or CT-guided drainages and may represent a less invasive, safe and effective strategy in the management of intra-abdominal and extra-peritoneal abscesses in selected patients. The principal cause of failure of percutaneous drainage is the misdiagnosis of the magnitude, extent, complexity, and location of the abscess.

In the setting of IAIs, the primary goals of the surgical intervention include: (a) determining the cause of the infection; (b) draining fluid/pus collections; (c) controlling the origin of the infection.

In patients with IAIs, surgical source control can include resection or suture of a diseased or perforated viscus (e.g., diverticular perforation, gastroduodenal perforation), removal of the infected organ (e.g. “Appendix”, gallbladder), debridement of necrotic tissue, resection of ischemic bowel, and repair/resection of traumatic perforations with primary anastomosis or creation of a stoma.

Source control does not only reduce bacterial and toxin load by removing the focus of infection and ongoing contamination but also transforms the local environment, hindering further microbial growth and optimizing host defences [19, 20].

Several research studies on patients with IAIs have consistently shown that failure to obtain adequate source control is one of the most important factors associated with adverse outcomes, including death [21, 22]. However, defining “adequacy” in source control remains a debatable term. Data collected from CIAO and CIAOW studies, in which a part of the enrolled cIAIs patients underwent surgical procedures to guarantee adequate source control, suggest that delays in the surgical treatment (> 24 h) represent an independent mortality predictor [23, 24]. However, in cases of uncomplicated IAIs, such as uncomplicated acute appendicitis, scheduling an appendectomy within 24 h from the diagnosis does not pose a higher risk of appendiceal perforation when compared to scheduling the procedure within 8 h [25].

Recently, multi-society guidelines for source control in the emergency setting were published [26]. The authors suggest that the initial assessment of patient stratification is a crucial first step in controlling the source of infection, and should consider the patient’s current health condition, comorbidities, and ongoing therapies (such as anticoagulants or steroids), as well as their immunological status. Patients can thus be categorized into three classes [26]:

1. Healthy patients with no or well-controlled comorbidities and not immunocompromised, where the infection is the major problem.
2. Patients with major comorbidities and/or moderately immunocompromised but clinically stable, in whom the infection can rapidly worsen the prognosis.
3. Patients with important comorbidities in advanced stages and/or severely immunocompromised, in which the infection deteriorates the pre-existing severe clinical condition.

The level of treatment urgency is determined by the affected organ(s), the rate at which symptoms develop, and the underlying physiological stability of the patient. In patients with IAIs source control should be rapidly achieved based on the patient’s clinical condition. The time between admission and initiation of the surgical procedure for source control is a critical and determinant factor influencing survival in patients with sepsis or septic shock of abdominal origin [27]. According to the current evidence, there is no reason to postpone source control for more than 6 h in patients with sepsis or septic shock of abdominal origin [28–31].

Some patients are prone to persisting signs of infection. In these patients, timely re-laparotomy provides an important surgical option that may significantly improve the clinical outcome when a single operation may not achieve an effective source control; thus, re-laparotomy may become necessary [32]. In adults with IAIs, on-demand re-laparotomy should represent the first choice of treatment.

Surgical strategies following an initial emergency laparotomy include subsequent “re-laparotomy on demand” (when required by the patient’s clinical condition) as well as planned re-laparotomy in the 36–48 h post-operative period. An on-demand laparotomy is performed only when the patient’s conditions deteriorate or do not improve and when CT scan findings show a benefit from additional surgery. Planned re-laparotomy is performed every 36–48 h for inspection, drainage, and peritoneal lavage of the abdominal cavity. A randomized trial published in 2007 by Van Ruler et al*.* [33] compared the differences between on-demand and planned re-laparotomy strategies in patients with severe cIAIs. Patients in the on-demand re-laparotomy group did not have a significantly lower rate of adverse outcomes compared with patients in the planned re-laparotomy but had a substantial reduction in re-laparotomies, healthcare utilization, and medical costs.

However, accurate and timely identification of patients needing a re-laparotomy is a very difficult decision-making process. At present, there are no clinical criteria to select patients for a re-laparotomy. Several studies have evaluated clinical variables that may be associated with the need for on-demand re-laparotomy in the immediate postoperative period [34–36], without defining standardized criteria.

The open abdomen may seem a viable option for treating physiologically deranged patients with ongoing sepsis, facilitating subsequent exploration and control of abdominal contents, and preventing abdominal compartment syndrome. However, there is still debate about the role of the OA in the management of patients with cIAIs.

### Haemodynamic support

Some patients with IAIs may present with sepsis. Sepsis and septic shock can be time-dependent emergencies. Early treatment with aggressive haemodynamic support can limit the damage of sepsis-induced tissue hypoxia and prevent the overstimulation of endothelial activity. Fluid resuscitation increases cardiac output, especially during the early stages of sepsis [37], and increases microvascular perfusion in patients with septic shock, leading to an improvement in organ function [38].

Starting fluid resuscitation as early as possible in the treatment of sepsis is necessary to compensate for capillary leakage of intravascular fluids, drains, gastrointestinal and insensible fluid loss [39, 40]. Although a protocolized resuscitation aimed at normalizing pre-defined physiological variables is no longer recommended [41–43], 30 mL/Kg of crystalloids in the first 3 h from recognizing sepsis and septic shock, irrespective of patients’ fluid status, patients’ comorbidities (e.g., heart failure, end-stage renal disease) and infection site [44] are always suggested. Because of the increased permeability of microcirculation and transcapillary leakage, fluid requirements in septic patients may be significant even after the completion of the initial resuscitation phase. This implies the need for further fluid boluses, in addition to maintenance fluids (including enteral fluids, feeding and parenteral nutrition) [45, 46]. In patients requiring large amounts of crystalloids to maintain cardiac output and peripheral perfusion, albumin solutions should be considered [44]. Despite the lack of a clear cut-off value for total crystalloids infused, data from a randomized controlled trial of septic patients showed that net fluid balance may be lower when 20% albumin is added to maintenance fluids. Albumin infusion may provide a survival benefit in the most severe group of septic patients (septic shock patients) [47, 48].

In addition to the clinical improvement (urine output > 0.5 mL/Kg/h, reduction of mottling, normalization of capillary refill time), current evidence supports the use of lactate clearance and serum lactate reduction as reliable indicators of resuscitation adequacy after volume loading and cardiac output restoration in septic shock patients [49].

In patients with abdominal sepsis, fluid resuscitation should be kept under control to avoid fluids overload. A recent meta-analysis of observational studies showed fluid overload was associated with mortality in patients with both acute kidney injury and surgery. Cumulative fluid balance was linked to mortality in patients with sepsis, acute kidney injury, and respiratory failure. The mortality risk increased by a factor of 1.19 (95% CI 1.11–1.28) per litre increase in positive fluid balance [50]. The haemodynamic consequences of intravascular congestion and the adverse effects of tissue oedema explain why fluid overload may lead to worse outcomes. Therefore, rather than infusing predefined amounts of fluids, the goal should be individualized fluids administration for every patient, based on evaluating both the need for fluids and the patient’s premorbid conditions [51].

Especially in patients with abdominal sepsis requiring urgent surgical intervention, aggressive fluid resuscitation improving the intravascular volume status and the organ perfusion may also increase intra-abdominal pressure (IAP) and worsen the inflammatory response, which is associated with a higher risk of complications. Several factors including systemic inflammatory response syndrome, increased vascular permeability and aggressive crystalloid resuscitation predisposing to fluid sequestration can result in peritoneal fluid formation and bowel oedema. Patients with advanced sepsis commonly develop bowel shock, resulting in excessive bowel oedema. These changes associated with forced closure of the abdominal wall may cause increased IAP, ultimately leading to intra-abdominal hypertension (IAH) [52]. IAP monitoring is a safe and cost-effective tool for identifying patients at risk for developing IAH and abdominal compartment syndrome (ACS) and can guide resuscitative therapy, reducing mortality and morbidity associated with IAH and ACS. Intra vesicular saline instillation is the most common technique to monitor IAP. It is a simple closed system that can measure bladder pressure in the ICU.

Vasopressor agents should be administered to restore organ perfusion as soon as possible if blood pressure is not restored after initial fluid resuscitation. Some evidence showed that early administration of vasopressors significantly increases shock control by 6 h [53, 54]. Norepinephrine is considered the first-line vasopressor agent to correct hypotension in patients with septic shock. Norepinephrine is a potent α-1 and β-1 adrenergic receptors agonist, which results in vasoconstriction and increased mean arterial pressure (MAP) with minimal effect on heart rate. Norepinephrine is more efficacious than dopamine and may be more effective in reversing hypotension in patients with septic shock. In a systematic review and meta-analysis [55], norepinephrine resulted in an absolute reduction of 11% in 28-day all-cause mortality when compared with dopamine. Dopamine was related to major adverse events, including an increase in the risk for cardiac arrhythmias. The haemodynamic profile of norepinephrine was more favourable than the other vasopressors, resulting in decreased lactate levels, increased central venous pressure and urine output compared to other vasopressors. Although most patients show a significant improvement in haemodynamic parameters after starting norepinephrine infusion, there is a remarkable proportion of septic shock patients with a poor clinical response to catecholamines, e.g. requiring large doses (> 0.5 mcg/Kg/min of norepinephrine) to reach MAP of 65 mmHg, or not reaching the threshold MAP, despite high-dose vasopressors and optimized fluid therapy [56]. In these cases, second-line vasopressors may provide some advantages instead of increasing norepinephrine infusion.

Vasopressin is an endogenous peptide hormone. It is produced in the hypothalamus and stored and released by the posterior pituitary gland. Its mechanism for vasoconstrictive activity is multifactorial and includes binding of V1 receptors on vascular smooth muscle resulting in increased arterial blood pressure. Vasopressin concentration is elevated in early septic shock but generally decreases to the normal range between 24 and 48 h as the shock continues. Studies have shown that the use of low-dose vasopressin (between 0.03 and 0.06 UI/min continuous infusion) reduces mortality in less severe cases of septic shock and has a "catecholamine-sparing" effect by decreasing the norepinephrine dose when both vasopressors are utilized [57, 58]. Thus, if patients require a norepinephrine dose > 0.25 mcg/Kg/min, adding vasopressin might be appropriate. When using vasopressin, it is advised to exercise caution due to the potential for limb extremities ischemia.

Epinephrine’s action is dose-dependent, having potent β-1 adrenergic receptor activity and moderate β-2 and α-1 adrenergic receptor activity. The activity of epinephrine, at low doses, is primarily driven by its action on β-1 adrenergic receptors, resulting in increased cardiac output, decreased systemic vascular resistance (SVR) and variable effects on MAP. At higher doses, however, epinephrine administration results in increased SVR and cardiac output. Studies have shown epinephrine continuous infusion can increase serum lactate levels regardless of tissue hypoperfusion [59]. In severe shock epinephrine can impair splanchnic circulation carrying a risk of splanchnic vasoconstriction and mesenteric ischemia [60].

Using corticosteroids in septic shock has been debated for decades. Corticosteroids increase vascular tone and catecholamine sensitivity [61] and their use has been advocated in patients with septic shock and relative adrenal insufficiency [62]. More recently, results of two large randomized controlled trials support stress-dose (200 mg/day) hydrocortisone administration in patients receiving moderate-to-high doses of norepinephrine (> 0.25 mcg/Kg/min). Although these studies failed to show a reduction in 28-day mortality, corticosteroids provided significantly faster resolution of shock and more rapid weaning from mechanical ventilation without increasing the infection rate [63, 64]. The reduction in vasopressor dose may be appealing, particularly in patients with septic shock because of cIAIs at risk of mesenteric ischemia.

### Infection prevention and control

Strengthening infection prevention and control practices along with implementing antimicrobial stewardship aims to optimizing antimicrobial use and improve patient outcomes, reduce antimicrobial resistance (AMR), and decrease the spread of infections caused by multidrug-resistant (MDR) organisms.

Infection prevention and control includes essential measures to reduce the incidence of healthcare-associated infections (HAIs) and prevent the emergence and spread of AMR. Enhancing hospitalized patient safety necessitates a systematic approach to preventing HAIs and AMR, because HAIs are frequently caused by MDR organisms. The occurrence of HAIs such as surgical site infections (SSIs), catheter-associated urinary tract infections (CA-UTIs), central line-associated bloodstream infections (CLA-BSIs), ventilator-associated pneumonia (VAP), hospital-acquired pneumonia (HAP), and *Clostridioides difficile* infection continues to escalate at an alarming rate.

Surgical patients may present several risk factors for the acquisition of HAIs. SSIs are the most prevalent HAIs in the surgical setting, especially in patients with IAIs having contaminated or dirty surgical fields [65]. SSIs can cause adverse patient outcomes, including prolonged hospital stays and higher related morbidity and mortality. Therefore, integrating SSI prevention measures before, during, and after surgery is essential. The management of most critically ill surgical patients usually involves the use of invasive devices (e.g., endotracheal tubes, vascular and urinary catheters), predisposing patients to the development of additional HAIs.

Since a significant proportion of HAIs is considered preventable [66] by respecting simple evidence-based measures such as hand hygiene practices, the use of barrier precautions, and implementing bundles to prevent specific HAIs like CLA-BSIs and VAP, strengthening infection prevention and control practices are essential to prevent their development.

The pathogenetic role of MDR microorganisms' gut colonization and the value of active surveillance are areas of debate. Screening for carriage of carbapenemase-producing *Enterobacterales* (CPE) is considered an important infection prevention tool, useful for control strategies [67, 68]. Some evidence suggests that a patient’s risk for CPE colonization should be personalized and assessed according to the local prevalence, individual risk of multi-drug-resistant pathogen acquisition, and linkages with other healthcare providers. Screening for carriage of CPE at admission is highly recommended for patients who within the last 12 months: (1) have been identified as carriers or have had a CPE related-infection, (2) have been hospitalized, (3) have received multiple antibiotic treatments, (4) have a known epidemiological link to a confirmed carrier of CPE, (5) are admitted to augmented care or high-risk units, or (6) have a planned major surgical abdominal intervention (e.g. solid organ transplantation) and/or have been exposed to immunosuppressive treatment (e.g. inflammatory bowel disease).

### Antimicrobial therapy

Empirical antibiotic therapy plays a crucial role in the effective management of IAIs; as inadequate initial antimicrobial treatment is associated with less favourable patient outcomes and the emergence of AMR, it is crucial to prescribe antibiotics correctly, with the right spectrum of activity, at the right time, for the right duration and with the right dosage. Optimizing antibiotic prescribing in the hospital setting results in improved treatment effectiveness and patient safety. This minimizes the risk of opportunistic infections such as *Clostridioides difficile* infection and mitigates the risk of selecting antimicrobial-resistant bacteria. The growing emergence of MDR organisms has caused an impending crisis with alarming implications, particularly concerning Gram-negative bacteria. Antimicrobial treatment should be started when a treatable infection has been recognized or strongly suspected. Misuse and abuse of antimicrobial agents, combined with the inappropriate application of infection prevention and control measures, are recognized as major drivers of the increasing prevalence of AMR.

AMR has become a global threat to public health systems in recent decades. Italy is ranked among the lowest-performing countries in AMR control in Europe by the European Centre for Disease Prevention and Control (ECDC), primarily due to alarmingly high levels of AMR observed in Italian hospitals [69]. In January 2017 a team of experts in antimicrobial stewardship selected by the ECDC planned a four-day visit to Italy to investigate and evaluate the situation in the country regarding prevention and control of AMR [70]. In the report drafted by the ECDC Committee, the experts highlighted the threat represented by the AMR, and the crucial necessity to design a national plan of action to address this burden.

In 2022, the ECDC published an interesting document evaluating the health impact of infections caused by antibiotic-resistant bacteria in the EU/EEA. The report, covering the period from 2016 to 2020, showed that the overall burden of infections attributed to AMR pathogens, adjusted for population size, was highest in Greece, Italy, and Romania [71]. In Italy, an alarming pattern of resistance involving MDR and extensively drug-resistant Gram-negative bacteria has emerged in recent years, and multi-resistant *Enterobacterales* are now a major concern in daily clinical practice. This phenomenon may be partially attributed to a high average age of the population, predisposing to the development and spread of AMR. However, it is likely to be influenced by a poor perception of the AMR burden. Hence, there is a critical need to raise awareness among Italian healthcare workers regarding the importance of the management of infections, including IAIs.

## Methodology

The Multidisciplinary and Intersociety Italian Council for the Optimization of Antimicrobial Use promoted a consensus conference on the antimicrobial management of IAIs, including emergency medicine specialists, radiologists, surgeons, intensivists, infectious disease specialists, clinical pharmacologists, hospital pharmacists, microbiologists and public health specialists.

Relevant clinical questions were constructed by the Organizational Committee in order to investigate the topic. The expert panel produced recommendation statements based on the best scientific evidence from PubMed and EMBASE Library and experts’ opinions.

The statements were planned and graded according to the Grading of Recommendations Assessment, Development and Evaluation (GRADE) hierarchy of evidence. The quality of the overall body of evidence has been defined as high, moderate, low, or very low. The strength of the recommendation was defined as weak or strong. For each statement and each algorithm, a consensus among the panel of experts was reached using a Delphi approach. Statements reaching an agreement of ≥ 80%, were approved as strong recommendations. On November 10, 2023, the experts met in Mestre (Italy) to debate the statements. After the approval of the statements, the expert panel met via email and virtual meetings to prepare and revise the definitive document. The manuscript was subsequently reviewed by all members who approved the present manuscript.

This document represents the executive summary of the consensus conference and comprises three sections. The first section focuses on the general principles of diagnosis and treatment of IAIs. The second section provides twenty-three evidence-based recommendations for the antimicrobial therapy of IAIs. The third section presents eight clinical diagnostic-therapeutic pathways for the most common IAIs. The document has been endorsed by the Italian Society of Surgery.

## Italian multidisciplinary Consensus Conference for the antimicrobial treatment of Intra-abdominal Infections

*What is the optimal timing to start antibiotic therapy in patients with IAIs?*

**1. In patients with IAIs, empiric antibiotic therapy should be started after a treatable infection has been recognized or highly suspected. Timing of administration should be based on the patient’s clinical status (Moderate quality of evidence, strong recommendation).**

Antibiotic therapy in patients with IAIs is initially empiric because the identification of pathogens and their susceptibility patterns determined by the standard microbiological culture typically needs 24 h or more.

Timing of administration should be based on the patient’s clinical status. In non-critically ill patients, empiric antimicrobial therapy should be started when an IAI is recognized or highly suspected. Survival benefit from adequate empiric antibiotic therapy has not been consistently shown, even in patients with Gram-negative bacteraemia [72]. Conversely, in patients with sepsis or septic shock, the prompt administration of appropriate empiric antibiotic therapy can significantly influence the outcome, regardless of the anatomical site of infection.

According to the current literature, a strong correlation exists between each hour of delayed administration of appropriate antibiotic therapy and mortality in patients with septic shock. However, this relationship is less pronounced in patients with sepsis who do not experience shock [73, 74].

*When should microbiological cultures be obtained in patients with IAIs?*

**2. In patients with IAIs at risk of resistant pathogens and in critically ill patients’, cultures from peritoneal fluid should be always obtained. In critically ill hospitalized patients, a minimum of two sets of blood cultures before initiating antimicrobial therapy should be always obtained (Very low quality of evidence, strong recommendation).**

Microbiological testing results are crucial in choosing a therapeutic strategy and in guiding a targeted antibiotic treatment. This testing allows clinicians to individualize the spectrum of the antibiotic regimen, broadening it if the initial choice was too narrow, but more commonly narrowing an empiric regimen spectrum that was too broad.

While microbiological cultures have limited influence on common community-acquired IAIs (CA-IAIs) such as acute appendicitis [75, 76], in the current era marked by the widespread circulation of MDR bacteria in both hospital and community settings, the burden of AMR cannot be disregarded. Microbiological testing is very important in managing hospital-acquired IAIs, where the risk of resistant pathogens is high. Antibiotic therapy reassessment based on microbiologic culture and susceptibility testing is suggested in critically ill patients. In critically ill hospitalized patients, the expert panel recommends obtaining a minimum of two sets of blood cultures before initiating antimicrobial therapy. It can improve diagnostic sensitivity, as it revealed a pathogen in more than half of the patients in which blood cultures were performed [77, 78]. These findings are notably higher compared to the 6% observed by Montravers et al*.* [79]. Microbiological testing is of great importance not only to define the therapeutic strategy for patients at risk of AMR but also to allows the clinician to better know the local epidemiology.

*Is antibiotic therapy reassessment based on the results of microbiological culture and susceptibility testing needed in patients with IAIs?*

**3. In patients with IAIs antibiotic, therapy should be reassessed as soon as possible when the results of microbiological culture and susceptibility testing are available (Moderate quality of evidence, strong recommendation).**

Microbiological diagnosis is crucial, because it may help clinicians to prescribe the most appropriate empiric antimicrobial therapy. Simultaneously, recognising the specific pathogen causing the infection can guide personalized and targeted antimicrobial therapy.

Microbiological cultures represent the gold standard methods for a correct microbiological diagnosis. Unfortunately, they are burdened by extended turnaround times. Delaying in starting an appropriate antimicrobial treatment has been associated with poorer patient’ outcomes, especially in patients with sepsis and septic shock [80–85]. As emphasized in the meta-analysis by Bassetti et al*.*, the use of an inadequate antibiotic regimen has been strongly associated with unfavourable outcomes in critically ill patients (OR 0.44, 95% CI 0.38–0.50), and increased length of hospital stay, and hospital costs [86].

The need to speed up diagnostic testing results is a central theme in recent policy initiatives to combat sepsis. Clinical microbiology is currently undergoing radical changes in diagnosing bloodstream infections (BSIs). Compared to conventional methods, fast clinical microbiology techniques can analyse microbiological samples accurately, identify pathogens along with the possible presence of major resistance genes, leading to the rapid confirmation of clinical suspicions of sepsis and warranting an early switch to a targeted antibiotic therapy. By applying these new methods to the diagnostic workflow of septic patients, the administration of appropriate antibiotics can promptly start, resulting in better clinical outcomes and decreased mortality [87–91].

A promising automated assay has recently been developed for IAIs, which can simultaneously identify a large panel of pathogen species (including both bacteria and fungi), toxins genes, and antibiotic resistance markers directly from intra-abdominal-specific solid and liquid samples [92]. A total of 300 clinical samples were tested with this technique by Ciesielczuk et al*.*, showing an overall sensitivity of 89.3% and specificity of 99.5%. When compared to standard methods, turnaround time in pathogen identification and full antibiotic susceptibility testing was reduced respectively by an average of about 17 and 41 h [92].

Although this promising premise requires more evidence, this panel suggests that fast microbiological methods should play a supportive role in acquiring a microbiological diagnosis and not substitute the actual gold standard method (standard culture).

*What is the optimal duration of antibiotic therapy in patients with IAIs?*

**4. IAIs should represent a not-to-miss opportunity for antimicrobial stewardship interventions. When adequate source control has been achieved, the antibiotic treatment duration can be substantially shortened (Moderate quality of evidence, strong recommendation).**

**5. In patients with uncomplicated acute appendicitis, laparoscopic appendectomy should be the standard treatment. If source control is adequate, it is unnecessary to administer post-operative antibiotic therapy to patients with uncomplicated acute appendicitis (Moderate quality of evidence, strong recommendation).**

**6. Antibiotic therapy alone can be performed in selected patients with uncomplicated acute appendicitis without appendicolith, advising for the possibility of high recurrence rates and misdiagnosing of complicated appendicitis (High quality of evidence, strong recommendation).**

**7. In patients with uncomplicated acute cholecystitis, laparoscopic cholecystectomy should be performed no later than 7 days from presentation. If source control is adequate, it is unnecessary to administer post-operative antibiotic therapy to patients with uncomplicated acute cholecystitis (Moderate quality of evidence, strong recommendation).**

**8. Early laparoscopic cholecystectomy can result in shorter hospitalization time and shorter courses of antibiotic therapy compared to delayed laparoscopic cholecystectomy. If early cholecystectomy is not performed, delayed cholecystectomy should be planned 6 and 12 weeks after acute cholecystitis (Moderate quality of evidence, strong recommendation).**

**9. In immunocompetent patients with uncomplicated acute diverticulitis antibiotic therapy may not be prescribed (Moderate quality of evidence, weak recommendation).**

A simple classification divides IAIs into complicated and uncomplicated. Uncomplicated IAIs are characterized by single-organ involvement and do not extend to the peritoneum. When the source of infection is treated effectively by surgical excision, post-operative antimicrobial therapy is unnecessary, as shown in the management of uncomplicated acute appendicitis or cholecystitis [93–95].

While appendicectomy represents the gold standard treatment for acute appendicitis, in recent years there has been a significant increase in the utilization of antibiotic therapy as a primary treatment method. Antibiotic therapy is a safe and effective primary treatment option for patients with uncomplicated acute appendicitis without an appendicolith. However, the long-term effectiveness of this approach is compromised by notable recurrence rates and poses a risk of perforation that may increase with preoperative delay, when a precise CT scan diagnosis of uncomplicated appendicitis has not been carried out [96, 97]. Hence, conservative treatment should be reserved for selected patients, while surgery represents the standard of care.

For acute cholecystitis, treatment is predominantly surgical. Two approaches are available to treat acute uncomplicated cholecystitis: the early option within a few days of onset of symptoms includes laparoscopic cholecystectomy to provide immediate, definitive surgical treatment after establishing the diagnosis and surgical fitness of the patient in the same hospital admission, while the delayed treatment option is performed in a second hospital admission after 6–12 weeks during which the acute inflammation settles [98]. In this setting, the role of antibiotic treatment is less defined than in acute appendicitis. However, it is short in early surgery, and longer in delayed surgery.

A systematic review and meta-analysis, including 15 randomized controlled trials (RCTs) and 1669 patients was published by Lyu et al*.* [99]. Early laparoscopic cholecystectomy resulted as safe and effective as delayed cholecystectomy for acute cholecystitis within 7 days from the presentation. No significant differences between the two approaches were found regarding bile duct injuries, wound infections, total complications, or conversion to open surgery. However, the pooled results showed that early surgical chelecystectmoy was related to a significantly shorter duration of hospital stay, without significant difference in postoperative hospital stay. A meta-analysis published in 2021 did not confirm that immediate cholecystectomy performed within 24 h of admission may reduce post-operative complications [100]. Importantly, the analysis of literature [101] showed that the timing of early cholecystectomy differed from cholecystectomy performed as soon as possible within 24 h of admission or up to 1 week of the onset of symptoms. Evidence has validated the window of 7 days from the presentation to perform an early cholecystectomy [101].

In recent years, there has been a debate about the need for antibiotic therapy in acute uncomplicated diverticulitis. In 2015, the World Society of Emergency Surgery (WSES) Acute Diverticulitis Working Group proposed a straightforward CT-guided classification of left colon acute diverticulitis. This classification aims to guide clinicians in the day-to-day management of acute diverticulitis and may be universally accepted. The WSES classification divides acute diverticulitis into 2 groups: uncomplicated and complicated. In the event of uncomplicated acute diverticulitis, the infection does not involve the peritoneum; whilst in complicated acute diverticulitis, the infective process extends beyond the colon [102].

In recent years, several studies showed that in patients with mild uncomplicated diverticulitis, antibiotic treatment was not superior to conservative treatment without antibiotics in terms of clinical resolution. The current consensus is that, in immunocompetent patients, uncomplicated acute diverticulitis may be a self-limiting condition in which local host defences can eradicate the inflammation process without antibiotics. Chabok et al*.* in 2012 published a multicenter randomized trial involving the joint participation of ten surgical departments in Sweden and one in Iceland [103]. A total of 623 patients with computed tomography-confirmed acute uncomplicated left-sided diverticulitis were enrolled and randomized to treatment with (314 patients) or without (309 patients) antibiotics. In this study, antibiotic treatment for acute uncomplicated diverticulitis neither sped up recovery nor prevented complications or recurrence. Therefore, antibiotics should be reserved to treat complicated diverticulitis. A prospective, cohort study [104] evaluated the safety and efficacy of non-antibiotic treatment for patients with CT-proven uncomplicated acute diverticulitis during a 30-day follow-up period. Overall, 161 patients were enrolled in the study, and 153 (95%) completed the 30-day follow-up. A total of 14 (9%) patients presented at the admission with pericolic gas. Altogether, 140 (87%) patients were treated as outpatients, and 4 (3%) of them were admitted to the hospital during the follow-up. None of the patients developed complicated diverticulitis or required surgical intervention, but 2 days (median) after inclusion, antibiotics were given to 14 (9%, 6 orally, 8 intravenously) patients. A recent Dutch randomized controlled trial of observational *versus* systemic antibiotic treatment (DIABOLO trial) [105] for a first episode of CT-proven ALCD Hinchey stages 1a and 1b confirmed that observational treatment without antibiotics did not prolong recovery and could be appropriate in these patients.

**10. In patients with cIAIs the duration of antibiotic therapy should be significantly shortened, based on the adequacy of source control (High quality of evidence, strong recommendation).**

**11. In patients with cIAIs, undergoing an adequate source control, 4 days fixed-duration antibiotic therapy should be administered. In the setting of complicated acute appendicitis, the duration of antibiotic therapy may be further shortened in selected patients (High quality of evidence, strong recommendation).**

**12. Patients who have ongoing signs of infection or systemic illness after 7 days of antibiotic therapy should warrant a new diagnostic investigation and clinical re-evaluation (Low quality of evidence, strong recommendation).**

**13. Biomarkers such as PCT may guide antibiotic duration in patients with ongoing signs of infection, and act as a valuable tool to suspect a worse evolution and to plan a re-laparotomy (Low quality of evidence, weak recommendation).**

In the event of cIAIs, the infectious process proceeds beyond the organ into the peritoneum, causing either localized or diffuse bacterial peritonitis. Treatment of patients with cIAIs involves both source control and antibiotic therapy. Antibiotics can prevent local and hematogenous spread and reduce late complications.

The value of the short duration of the treatment in patients with cIAIs is well documented. In the setting of cIAIs, a short course of antibiotic therapy after adequate source control is considered a safe option. The prospective trial (STOP-IT) by Sawyer et al*.* [106] demonstrated that in patients with cIAIs undergoing an adequate source control, the outcomes after approximately 4 days of fixed-duration antibiotic therapy were similar to outcomes after a longer course of antibiotics extending until after the resolution of physiological abnormalities.

Probably in the setting of acute appendicitis, the duration of antibiotic therapy could be further shortened. The results of an open-label, non-inferiority trial enrolling patients with complex appendicitis (aged ≥ 8 years) investigating the duration of antibiotic therapy were published recently. Two days of postoperative intravenous antibiotics for complex appendicitis was non-inferior to 5 days in terms of infectious complications and mortality within 90 days, based on a non-inferiority margin of 7.5% [107].

Short courses of antibiotic therapy were also shown to be feasible in patients with post-operative peritonitis [108]. A multicenter prospective randomized trial conducted in 21 French ICUs between May 2011 and February 2015 compared the efficacy and safety of 8-day *versus* 15-day antibiotic therapy in critically ill patients with post-operative IAIs. Patients treated for 8 days had a higher median number of antibiotic-free days than those treated for 15 or 12 days (*p* < 0.0001). Equivalence was established in terms of 45-day mortality (rate difference 0.038, 95% CI 0.013–0.061). Treatments did not differ in terms of ICU and hospital length of stay, emergence of multi-drug-resistant bacteria, or re-operation rate. The trial showed that a short-course of antibiotic therapy in critically ill ICU patients with post-operative IAIs reduced antibiotic exposure. Continuation of treatment until day 15 was not associated with any clinical benefit.

Recently, a retrospective cohort study of 42 adult surgical ICU patients with BSIs secondary to IAIs was published. Cessation of antibiotic therapy within 7 days from adequate source control was not associated with an increased incidence of recurrence [109].

Considering the lack of generalizable data regarding the optimal duration of therapy for critically ill patients, there may be significant variations in practices in this setting. Many surgical critically ill patients often receive antibiotics for a longer duration than necessary. In patients with evidence of an ongoing infection, an individualized approach is mandatory. Clinical judgment associated with the patient’s inflammatory response monitoring by biomarkers trend (PCR and PCT) could empower any decision about continuing, narrowing, or stopping antibiotic therapy.

Patients who have persistent signs of infection or systemic illness after 7 days of antibiotic therapy warrant a second-level diagnostic re-investigation to determine whether additional surgical intervention or percutaneous drainage is necessary to address an ongoing uncontrolled source of infection or antibiotic treatment failure [110].

Patients enrolled in STOP-IT trial were evaluated retrospectively to identify risk factors associated with treatment failure [111]. This subgroup analysis was able to identify risk factors for treatment failure, including corticosteroid use, Acute Physiology and Chronic Health Evaluation II score ≥ 5, HAIs, or a colonic source of IAI [111]. However, among patients with these risk factors, there was no significant difference in treatment failure rates between the randomized groups. These results suggest that individuals at a high risk of treatment failure did not benefit from a longer duration of antibiotic therapy.

In recent years, PCT has been useful in individualizing antibiotic use duration. Evidence shows that this pro-inflammatory biomarker safely shortens antibiotic duration in critically ill patients in the ICU [112–116]. Some evidence showed the role of PCT in guiding the duration and/or cessation of antibiotic therapy in cIAIs. Three studies showed that a PCT-based algorithm could decrease antibiotic exposure in patients with cIAIs. Huang et al*.* [117] published in 2014 a prospective study investigating whether a PCT-based algorithm could safely reduce antibiotic exposure in patients with cIAIs undergoing surgery. PCT levels were obtained pre-operatively, on postoperative days 1, 3, 5, and 7, and on subsequent days if needed. Antibiotic treatment was discontinued if PCT was < 1.0 ng/L or decreased by 80% *versus* day 1, with a resolution of clinical signs. The PCT algorithm significantly improved the time to antibiotic discontinuation (*p* < 0.001, log-rank test). The median duration of antibiotic treatment in the PCT group was 3.4 days (interquartile range [IQR] 2.2 days), *versus* 6.1 days (IQR 3.2 days) in the control group. In 2015, Maseda et al*.* [118] published a retrospective study including 121 consecutive patients with cIAIs, a controlled infection source, requiring surgery, and at least 48-h surgical ICU admission. Treatment was shorter in the PCT-guided group (5.1 ± 2.1 *versus* 10.2 ± 3.7 days, *p* < 0.001), without differences between patients with and without septic shock. PCT guidance produced a 50% reduction in antibiotic therapy duration (*p* < 0.001, log-rank test). In 2017, Slieker et al*.* [119] published another study to investigate whether PCT levels could tailor postoperative antibiotic therapy in patients with cIAIs undergoing surgery. In the subgroup of patients with cIAIs caused by gastrointestinal perforation, the duration of antibiotic treatment was significantly reduced in the PCT-driven algorithm (7 days in the PCT group *versus* 10 days in the control group (*p* = 0.065).

*How should the correct antibiotic be chosen in patients with IAIs?*

**14. In patients with IAIs, empiric antibiotic therapy should be based on the local microbiological epidemiology, clinical severity, and individual patient risk factors for resistant bacteria (Low quality of evidence, strong recommendation).**

**15. In most patients with IAIs, agents with a narrow spectrum of activity should be preferred. In community-acquired IAIs, the most common resistance problem is posed by alarmingly prevalent extended-spectrum beta-lactamases (ESBLs). (Moderate quality of evidence, strong recommendation).**

**16. The following risk factors for ESBLs-producing Enterobacterales infections should be always considered: (a) hospitalization for 48 h within the last 90 days, (b) use of broad-spectrum antibiotics for 5 days within the last 90 days, (c) gut colonization by ESBLs within 90 days and (d) patients coming from healthcare settings with a high incidence of MDR bacteria (Low quality of evidence, strong recommendation).**

**17. Antibiotic therapy aimed at enterococcal coverage should not be routinely prescribed in patients with community-acquired IAIs unless they are immunocompromised (Moderate quality of evidence, strong recommendation).**

**18. Empirical antibiotic therapy covering MDR Gram-negative bacteria should be considered only in specific settings, and based on country-wide epidemiological conditions, clinical severity, immunological impairment, knowledge of colonization status, prolonged exposure to carbapenems and/or quinolones (Moderate quality of evidence, strong recommendation).**

In the setting of IAIs, inappropriate choice of initial antibiotic therapy in patients leads to more clinical failure, resulting in a longer hospital stay and higher costs of hospitalization compared with appropriate initial antibiotic therapy [120–123]. Before causative agent(s) and susceptibilities are known, the optimal choice of antibiotic therapy depends on the local prevalence of resistant bacteria and patient risk factors for them as long as available microbiological data (e.g. colonization status).

The major pathogens involved in CA-IAIs are likely to be due to a patient’s flora and are generally predictable and include *Enterobacterales* such as *Escherichia coli*, *Klebsiella* spp., viridans group streptococci, and anaerobes (especially *Bacteroides fragilis*). In addition, *Enterococcus* spp. are Gram-positive bacteria frequently isolated in CA-IAIs, even if their pathogenic role remains uncertain [124]. In 2012, the Dutch Peritonitis Study Group analysed all patients from the RELAP trial and found that the isolation of Gram-positive cocci [125], predominantly *Enterococcus* spp., was associated with worse outcomes, although in cIAIs, microbial profiles did not predict ongoing abdominal infection after initial emergency laparotomy [126].

Generally, the key factors for predicting the presence of resistant bacteria in patients with cIAIs include acquiring the infection in a healthcare setting, recent antibiotic therapy, prior infection by MDR bacteria and gut colonization [127]. Patients with post-operative peritonitis have increased mortality due to the severity of the clinical condition, underlying comorbidity, frequent atypical presentation, and significant incidence of acquiring resistant bacteria.

Over the past two decades, AMR has emerged as a global burden. The rise in infections caused by resistant Gram-negative bacteria poses an escalating threat to public health worldwide. These infections are challenging to treat and are associated with elevated morbidity and mortality rates. To identify the risk factors for resistant bacteria in post-operative peritonitis, Augustin et al*.* conducted a review of data from 100 patients hospitalized in the ICU. Logistic regression analysis revealed that the use of broad-spectrum antibiotics between the initial intervention and re-operation was the sole significant risk factor for the emergence of resistant bacteria [128]. In a retrospective study, data from 242 patients with cIAIs (88 community-acquired, 154 post-operative cases) treated in the ICU were obtained [129]. *Enterococci* were isolated in 47.1% of all patients, followed by *E. coli* (42.6%), other *Enterobacterales* (33.1%), anaerobes (29.8%), and *Candida* spp. (28.9%). The susceptibility rates were lower in post-operative than in community-acquired cases.

The epidemiology of intra-abdominal flora in critically ill patients with secondary and tertiary abdominal sepsis was studied by a 5-year prospective observational cohort study [130], performed in patients admitted to the ICU with abdominal sepsis syndrome. Abdominal fluid samples for microbiological analysis were collected from 221 of the 239 patients admitted with abdominal sepsis. Aerobic Gram-negative bacteria (AGNB) were isolated in 53% of the cultures of the abdominal fluid. Among them, 45% were *E. coli*; in 36% of patients, more than one AGNB was found. The highest incidence of AGNB was observed in colorectal perforations (68.6%) and perforated appendicitis (77.8%), while the lowest incidence was observed in gastroduodenal perforations (20.5%). Gram-positive bacteria were found most frequently in colorectal perforations (50.0%). *Candida* spp. was found in 19.9% of patients, with 59.1% of isolates represented by *Candida albicans*. The incidence of *Candida* was higher in gastroduodenal perforations (41%) and lower in colorectal perforation (11.8%). Anaerobic bacteria were cultured in 77.8% of patients with perforated appendicitis. Montravers et al*.* [131] evaluated the dynamic change of microbial flora in persistent peritonitis and observed a progressive shift of peritoneal flora with the number of reoperations. The proportion of patients harbouring MDR strains increased from 41% at index surgery to 49% at the first, 54% at the second (*p* = 0.037), and 76% at the third re-operation (*p* = 0.003 *versus* index surgery), highlighting the necessity and utility of collecting intraperitoneal specimens in every re-intervention.

In 2019 Maseda et al*.* [132] published a one-year prospective observational study from 17 Spanish ICUs distributing cases of healthcare-associated infections (HCAIs), CA-IAIs and immunocompromised patients. Bacteria producing ESBLs and/or CPE, high-level aminoglycoside- and/or methicillin- and/or vancomycin- resistance were considered AMR. Mortality-associated factors were identified by multivariate regression analysis. Of 345 patients included, 51.6% presented generalized peritonitis; 32.5% were > 75 years. Overall, 11.0% of cases presented AMR (7.0% ESBLs- and/or CPE), being significantly higher in HAIs (35.4%) *versus* CA-IAIs (5.8%) (*p* < 0.001) *versus* immunocompromised patients (0%) (*p* = 0.003). Overall, the 30-day mortality was 14.5% (23.1% for HAI and 11.6% for CA-IAIs; *p* = 0.016) and was positively associated with age > 75 years, *Candida* isolation, and SAPS II level. A lower mortality rate was observed in biliary infections.

In both community- and hospital-acquired IAIs, the most common resistance threat is posed by ESBLs, which are becoming alarmingly prevalent also in the community setting [133, 134]. ESBLs are enzymes capable of hydrolysing and inactivating a wide variety of beta-lactam drugs, including first-, second-, and third-generation cephalosporins, penicillins, and aztreonam. ESBLs are not effective against cephamycins and carbapenems [135, 136]. Most ESBLs of clinical interest are encoded by genes located on plasmids, therefore resistance to other multiple-drug classes including aminoglycosides and fluoroquinolones may be co-expressed [136].

The main risk factors for ESBLs-producing infections are: (1) hospitalization for at least 48 h within the last 90 days, (2) use of broad-spectrum antibiotics for 5 days within the last 90 days, (3) gut colonization by ESBL within 90 days, (4) patients coming from healthcare settings with high incidence of MDR bacteria (e.g. elderly people living in long-term facilities) [137]. According to the latest annual report from the EARS-NET network of national surveillance systems across EU/EEA countries, the AMR situation in 2022 varied widely depending on the bacterial species and the geographical area. A latitude-dependent gradient in the prevalence of AMR was highlighted. Countries in northern Europe reported the lowest rates, while countries in southern and Eastern Europe reported the highest rates. In this report, high rates of third-generation cephalosporin-resistant *E. coli* have been found in Greece, Italy, Spain, and the eastern European Countries. The highest rates of third-generation cephalosporin-resistant *K. pneumoniae* have been reported in Italy, Greece, Croatia, Poland, Slovakia, Romania and Bulgaria [69]. However, the knowledge of the national epidemiology is not sufficient to accurately assess the patient's risk of ESBL-related infections. As highlighted in the latest AR-ISS report published by the Italian Antimicrobial Resistance Surveillance Agency [138], third-generation cephalosporin-resistant *E. coli* rates reported in 2022 vary significantly among the Italian regions, following a North–South gradient with the highest rates detected in Molise (42.6%), Calabria (39.9%), Campania (38.2%), Basilicata (35.2%), Sicily (35.15), Puglia (34.9%), and Lazio (31.8%). In all Northern regions, a prevalence lower than 26% is observed, with the lowest values in the Autonomous Province of Bolzano (10.9%) and Friuli-Venezia-Giulia (11.9%).

Carbapenems are generally considered the empiric agents of choice for treating patients with the most common ESBL-producing *Enterobacterales*. To avoid excessive carbapenem use, however, de-escalation to other agents, such as piperacillin-tazobactam when a MIC ≤ 8 mg/L (according to the EUCAST breakpoint) is detected, can be considered. Several studies compared piperacillin-tazobactam with carbapenems in the treatment of infections caused by ESBL-producing *Enterobacterales*. In the MERINO trial [139], the efficacy of piperacillin-tazobactam *versus* meropenem in the treatment of BSIs caused by ceftriaxone-resistant *E. coli* or *K. pneumoniae* was compared, showing an overall 30-day mortality rate threefold higher in the piperacillin-tazobactam arm than in the meropenem one (12.3% *versus* 3.7%, *p* = 0.90). Since the low mortality rate in the meropenem group was an unexpected finding, the results of this study have been debated and several issues may have influenced the outcomes of this trial [140, 141]. Among the debated biases, the pharmacokinetic/pharmacodynamic (PK/PD) target attainment of piperacillin-tazobactam was not optimized, as the trial favoured intermittent 1-h infusion over a prolonged infusion protocol. Also, from a microbiological point of view, the use of the E-test to determine piperacillin/tazobactam susceptibility led to an elevated percentage of OXA-1-producing pathogens being incorrectly identified as piperacillin-tazobactam susceptible (E-test method cannot detect OXA-1). In a second comparative study (MERINO-2 trial), 72 patients with BSIs due to chromosomal AmpC producers were enrolled in a multicenter randomized controlled trial, where they were assigned 1:1 to receive piperacillin-tazobactam or meropenem. Piperacillin-tazobactam led to more microbiological failures, although fewer microbiological relapses were observed [142]. Despite several pieces of evidence coming from observational studies showed no significant difference in efficacy and mortality rate between piperacillin-tazobactam and carbapenems among patients with ESBL-producing BSIs [143–146], the use of a carbapenem (imipenem or meropenem) for severe infections caused by third-generation cephalosporin-resistant *Enterobacterales* is generally recommended in critically ill patients [147].

Tigecycline remains a viable treatment option for complicated IAI due to its favourable in vitro activity against anaerobic organisms, *enterococci* and ESBLs, and to the high concentration achieved in the biliary tract [148]. However, in numerous trials, excess mortality was seen in patients treated with tigecycline when compared with other drugs; in 12 of 13 phase 3 and 4 comparative clinical trials [148], all-cause mortality was found higher in the tigecycline group *versus* the comparison group. Study-level and patient-level analyses identified that patients in the hospital-acquired pneumonia trial, particularly those with ventilator-associated pneumonia with baseline bacteraemia, were at a higher risk of clinical failure and mortality. A mortality analysis was used to investigate the association of baseline factors in intra-abdominal infections, including severity of illness at study entry and treatment assignment, with clinical failure and mortality. Mortality modelling identified multiple factors associated with death which did not include tigecycline [148]. Because of its high concentration in the biliary tract, despite its low performance in bacteraemia patients, tigecycline could be considered as an alternative to beta-lactam agents in the setting of IAIs, when considering a combination antibiotic treatment when a secondary bloodstream infection is suspected.

Aminoglycosides are particularly effective against aerobic Gram-negative bacteria and can act synergistically against certain Gram-positive organisms. They are active against *Pseudomonas aeruginosa*, but are ineffective against anaerobic bacteria. Because of their serious toxic side effects including nephrotoxicity and ototoxicity, and considering the poor penetration rate in the ascitic fluid and the loss of bactericidal activity in the presence of acidic pH, most authors do not recommend aminoglycosides for the routine empiric treatment of IAIs.

Fosfomycin is a broad-spectrum antibiotic with a wide therapeutic range and characteristic pharmacological properties [149]. Fosfomycin penetrates excellently into various tissues and is frequently administered in combination with other antibiotics to combat severe bacterial infections in Europe. It exerts bactericidal activity under anaerobic conditions, such as is the case within encapsulated purulent lesions, and has negligible protein binding. These characteristics constituted the rationale for choosing fosfomycin in treating abscesses if source control is not feasible.

Finally, ceftolozane-tazobactam and ceftazidime-avibactam (CAZ-AVI) have shown good efficacy in treating patients with IAIs caused by ESBL-producing *Enterobacterales*, mostly CAZ-AVI due to the higher enzymatic inhibition of avibactam [146]. They may be useful in patients with ESBL-*Enterobacterales* infections, as a part of carbapenem sparing regimens [148]. Due to their activity, CAZ-AVI and ceftolozane-tazobactam should not be used alone because they are not active against anaerobes and Gram-positive bacteria such as *Streptococci* and methicillin-susceptible *Staphylococcus aureus*.

Carbapenemase-producing *Enterobacterales* (CPE), such as *K. pneumoniae*, are rapidly emerging as a major source of MDR infections worldwide and pose a serious threat in clinical situations where administration of effective empiric antibiotics is essential to prevent mortality following bacteraemia and infections in neutropenic and immunocompromised patients. According to the latest annual report from the EARS-NET, high rates of *K. pneumoniae* carbapenemase (KPC) have been found in Greece, Italy, Portugal, Croatia, Poland, Romania and Bulgaria [69]. In Italy, the latest AR-ISS report reveals high percentages of KPC in the southern region with an important North–South gradient [highest in Calabria (59.2%), and lowest in the Autonomous Province of Bolzano (1.3%)] [138]. In the last five years, there have been several new antibiotics with predominant activity against Gram-negative bacteria approved by the U.S. Food and Drug Administration and the European Medical Agency.

Some prospective and several retrospective observational studies support CAZ-AVI in the treatment of BSIs, cIAIs, and complicated urinary tract infections in settings with an ICU admission for up to 60% [150–157]. Van Duin et al*.* [150] assessed prospectively 137 CPE infections (38 treated with CAZ-AVI *versus* 99 with colistin-based regimens). In patients treated with CAZ-AVI *versus* colistin, 30-day hospital mortality after starting treatment was 9% *versus* 32%, respectively. Moreover, at 30 days, patients treated with CAZ-AVI, compared with those treated with colistin, had a 64% probability of better outcomes. Tumbarello et al*.* demonstrated the effectiveness of CAZ-AVI against KPC-producing *K. pneumoniae* (KPC-Kp) infections by two retrospective observational studies, conducted in Italy [151, 152]. The first study enrolled 138 patients starting CAZ-AVI salvage therapy after first-line treatment (median, 7 days) with other antibiotics [151]. CAZ-AVI was administered with at least 1 other active antibiotic in 109 (78.9%) cases. Thirty-day mortality among the 104 patients with BSIs secondary to KPC-Kp infections was significantly lower than that of a matched cohort whose KPC-Kp bacteremia had been treated with drugs other than CAZ-AVI (36.5% *versus* 55.8%, *p* = 0.005). In the second study 577 adult patients with BSIs (391) or nonbacteremic infections, involving mainly urinary tract, lower respiratory tract and intra-abdominal structures, were analyzed [152]. All received treatment with CAZ-AVI alone (165) or with ≥ 1 other active antibiotics (412). The all-cause mortality rate 30 days after infection onset was 25% (146/577). There was no significant difference in mortality between patients managed with CAZ-AVI alone and those treated with combination regimens (26.1% *versus* 25.0%, *p* = 0.79). Only 35 out of 577 BSIs were associated with IAIs. This did not give sufficient information about the role of the drug in this specific setting but conformed how often IAIs are compartmentalized. CAZ-AVI has activity against most KPC and OXA-48-like-producing CPE, and currently, it represents the preferred treatment option for OXA-48-like-producing infections [158].

Meropenem-vaborbactam is another agent active against KPC-producing CPE. A phase III RCT (TANGO II) [159] assessed 47 patients affected by CPE infections. Of these patients, 32 were treated with meropenem-vaborbactam and the other 15 with best-available therapy (including mono/combination therapy with colistin, carbapenems, aminoglycosides, tigecycline, or ceftazidime-avibactam alone). Meropenem-vaborbactam showed a better clinical cure rate (65.6% *versus* 33.3%; *p* = 0.03) and a lower mortality rate (15.6% *versus* 33.3%; *p* = 0.20) compared to the best available therapy. However, patients enrolled in this RCT required ICU admission only in 15.6% of cases. Other evidence for meropenem-vaborbactam as targeted therapy for CPE infections in critically ill patients came from observational studies, in which ICU admission ranged from 65.4% to 70% [160, 161].

Imipenem-relebactam, another agent active against KPC, was compared to imipenem and colistin in the treatment of hospital-acquired bacterial pneumonia and ventilator-associated bacterial pneumonia, cIAIs, or complicated urinary tract infections caused by imipenem-non susceptible bacteria (RESTORE-IMI 1 trial) [162]. The favourable overall response was observed in 71% imipenem/relebactam and 70% colistin + imipenem patients. Day 28 favourable clinical response was observed in 71% imipenem/relebactam and 40% colistin + imipenem patients, and 28-day mortality in 10% imipenem/relebactam and 30% colistin + imipenem patients. Unfortunately, only 2 patients per arm with cIAIs were enrolled. Serious adverse events, as well as nephrotoxicity, occurred more often in patients treated with imipenem and colistin. Imipenem-relebactam retains good in vitro activity against *P. aeruginosa*. Of 1912 *P. aeruginosa* isolates recovered as a part of a multicenter Canadian surveillance study [163], 166 (8.7%) and 495 (25.9%) demonstrated difficult-to-treat resistance and MDR phenotypes, respectively. Among these isolates, several antibiotics were tested. Impenem-relebactam susceptibility was 47.0% for difficult-to-treat resistance isolates and 71.5% for MDR isolates, second only to ceftolozane-tazobactam, and better than CAZ-AVI.

Meropenem-vaborbactam and imipenem-relebactam are active against most *Enterobacterales* producing KPC enzymes but not those producing OXA-48-like carbapenemases [164, 165]. Neither ceftazidime-avibactam, meropenem-vaborbactam, nor imipenem-relebactam has activity against metallo-beta-lactamase-producing *Enterobacterales*. Therefore, all 3 of these agents are qualified options for CPE clinical isolates outside of the urinary tract.

However, the emergence of resistance to CAZ-AVI in CPE has been repeatedly reported. Several KPC variants have emerged, with changes (substitution, insertion, or deletion) in the amino acid sequence compared to wild-type KPC enzymes (e.g. KPC-2 and KPC-3), conferring reduced susceptibility or resistance to CAZ-AVI [166]. So far, more than 200 KPC variants have been reported worldwide, with several reports of resistance extended also to meropenem-vaborbactam and sometimes to imipenem-relebactam. These evidences increase the difficulties in optimizing infection treatment and preventing the emergence of new resistant phenotypes/genotypes [167, 168].

Eravacycline, a broad-spectrum fluorocycline tetracycline antibiotic, was investigated in the treatment of cIAI by two prospective randomized clinical trials in which a non-inferior clinical cure rate in eravacycline population at the test-of-cure visits was found when compared to ertapenem and meropenem (IGNITE 1 trial: 87.0% for eravacycline *versus* 88.8% for ertapenem; IGNITE 4 trial: 90.8% *versus* 91.2%) [169, 170]. Also, a very low risk of *C. difficile* infection after eravacycline treatment was observed [170, 171]. In a recent meta-analysis, Meng et al*.* revised the results of 25 randomized clinical trials to evaluate the efficacy and safety of eravacycline compared with other seven regimens commonly used for cIAIs treatment (tigecycline, meropenem, ertapenem, ceftazidime/avibactam + metronidazole, piperacillin/tazobactam, imipenem/cilastatin, and ceftriaxone + metronidazole). In terms of microbiological response rate, eravacycline was significantly better than tigecycline [tigecycline *versus* eravacycline: RR = 0.82, 95% CI (0.65,0.99)], and there was no significant difference between the other 6 regimens and eravacycline (*p* > 0.05). In terms of safety, the incidence of serious adverse events, discontinuation rate, and all-cause mortality of eravacycline were not significantly different from those of the other 7 treatment therapies (*p* > 0.05). [172]. Both the European Society of Clinical Microbiology and Infectious Diseases (ESCMID) and the Infectious Diseases Society of America (IDSA) guidance suggest using eravacycline as an alternative option for the treatment of infections secondary to ESBL-producing and CPE (including KPC, metallo-beta-lactamases [MBLs], OXA-48 producing strains), except for the treatment of bloodstream or urinary tract infections [173]. Finally, in Hobbs Real-World experience, a possible clinical efficacy of eravacycline in treating infections due to carbapenem-resistant *Acinetobacter baumannii* was hypothesized, even though further evidence is needed [171]. Like tigecycline, eravacycline presents large volume of distribution with excellent tissue penetration, which is supposed to limit its use in primary BSIs [174], and high biliary secretion. However, a post-hoc analysis conducted by Lawrence et al*.* eravacycline demonstrated a similar microbiological eradication rate as comparator agents in patients with cIAI associated with secondary bacteraemia [175].

Cefiderocol is a siderophore cephalosporin. It has shown excellent broad-spectrum antibacterial activity, in part due to its innovative way of cell entry. However, some mechanisms of resistance to cefiderocol have already been identified and reduced susceptibility has developed during patient treatment. Therefore, the clinical use of cefiderocol should be rational. In a phase 3 RCT 150 patients affected by carbapenem-resistant Gram-negative infections were randomized to cefiderocol (n = 101) or the best available therapy, including a combination of aminoglycoside, carbapenems, colistin, fosfomycin or tigecycline (n = 49) [176]. Clinical and microbiological cure rates did not significantly differ in the two groups. However, the number of documented CPE infections was limited and the number of patients with IAIs enrolled was low (5 in the cefiderocol arm and 4 in the best available therapy arm). Moreover, more deaths occurred in the cefiderocol group, primarily in the patient subset with *Acinetobacter* spp. infections. Several non-controlled studies were published about Cefiderocol afterwards [177–180]. Although, Cefiderocol represents a very useful therapeutic option, further clinical data are needed to better understand the role of this novel option in the extensive-drug resistant infection treatment scenario [181, 182].

MBLs differ structurally from the other beta-lactamases by their requirement for a zinc ion at the active site. They are all capable of hydrolysing carbapenems. In contrast to the serine beta-lactamases, the MBLs have poor affinity or hydrolytic capability for monobactams and are not inhibited by clavulanic acid, tazobactam, avibactam, relebactam and vaborbactam. The most common metallo-beta-lactamase families encountered in Gram-negative bacilli include the IMP (active-on-imipenem beta-lactamase), VIM (Verona integron-encoded metallo beta-lactamase) and NDM (New Delhi metallo-beta-lactamase) [148].

The lack of in vitro activity of ceftazidime-avibactam against MBLs and the fact that many MBLs producers also coproduce other beta-lactamases (such as ESBLs, AmpC, OXA-48, etc.) have led to hypothesize a potential effect of combining ceftazidime-avibactam with aztreonam, which is not hydrolysed by MBLs per se [183–186]. A real potential breakthrough in the treatment of MBLs could be represented by the recent development of a new antibiotic, made from the combination of the monobactam aztreonam with the non-beta-lactam β-lactamase inhibitor avibactam.

Aztreonam-avibactam is currently under clinical development for the treatment of serious infections caused by MBLs-producing *Enterobacterales* [181].

Alarming rates of resistance to many antibiotics in hospitals worldwide have been reported for non-fermenting Gram-negative bacteria, including *Pseudomonas aeruginosa*, *Stenotrophomonas maltophilia*, and *Acinetobcter baumannii*. These bacteria are intrinsically resistant to many antibiotics; moreover, they can acquire additional resistance to other important antibiotic agents.

Among Gram-positive bacteria involved in IAIs, the impact of *Enterococcus* spp. on mortality remains uncertain [187]. While the role of *Enterococci* spp. in determining breakthroughs or superinfections in high-risk patients is well documented, their pathogenic impact on IAIs in low-risk patients is still debated [188]. As far as *Enterococcus* spp. is believed to possess low virulence factors, it may be supposed that an increase in virulence may be obtained by the development of a synergistic effect with other bacteria like *E. coli* and anaerobes [187, 189]. Various observational studies highlighted the treatment failure of patients infected by *Enterococcus* spp. relates with a poorer outcome; however, there is no consistent evidence that routine use of adequate anti-enterococcal coverage improves the survival rate, hypothesizing that *Enterococcus* spp. isolation represents a negative prognostic marker rather than playing a causative role in the infection [190, 191]. In their meta-analysis, Zhang et al*.* [126] found that enterococci-covered antibiotic regimens provided no improvement in treatment success compared with control regimens (RR, 0.99; 95% CI, 0.97–1.00; *p* = 0.15), with similar mortality and adverse effects in both arms. Basic characteristic analysis revealed that most of the enrolled patients with IAIs in RCTs were young, lower-risk CA-IAIs patients with a relatively low APACHE II score. Interestingly, malignancy, corticosteroid use, surgical intervention, antibiotic treatment, admission to the ICU, and indwelling urinary catheters could predispose patients with IAI to a substantially higher risk of enterococcal infection. Also, the acquisition of an IAI in the hospital setting seemed to represent a risk factor for enterococcal infections (OR, 2.81; 95% CI, 2.34–3.39; *p* < 0.001) [126]. In Dupont et al*.* study [189], patients older than 75 years and admitted to ICU with *Enterococcus* spp. isolation presented higher SAPS2 and SOFA scores when compared with the *enterococci*-negative control group (*p* < 0.001 for both scores), confirming that the identification of these bacteria represents an independent risk factor for ICU mortality. Similarly, in Morvan et al*.* study ICU patients had higher 30-day mortality when *Enterococcus* spp. was isolated, especially when species other than *Enterococcus faecalis* (mostly *E. faecium*) or a polymicrobial infection were detected [187]. Accordingly, to the above-described evidence, Sanders et al*.* concluded that the only parameter able to predict *Enterococcus* spp. isolation was the APACHE-II score (unadjusted odds ratio [OR] 1.07; *p* < 0.01) [192]. In Fabre et al*.* multicentre study where approximately 65% of patients in both groups had CA-IAIs, there was no difference in the 30-day composite outcome between cIAI patients with *E. faecalis* isolation from intra-abdominal cultures and those treated with piperacillin/tazobactam rather than receiving ertapenem therapy (which cannot guarantee an adequate anti-enterococcal coverage) (OR 0.80; 95% CI; 0.39–1.63) [193].

An extensive literature review demonstrated some evidence in favour of using empirical therapy with enterococcal coverage for IAIs in the following cases: 1) immunocompromised patients or patients with hospital-acquired/post-operative cIAIs; 2) patients with cIAIs who have previously received cephalosporins and other broad-spectrum antibiotics selecting *Enterococcus* spp.; 3) patients with cIAIs and valvular heart disease or prosthetic intravascular material, at high risk of endocarditis. The ideal therapeutic regimen for these high-risk patients remains to be determined, but empirical therapy directed against *enterococci* should be considered [194].

Nearly all strains of *E. faecalis*, including some strains of vancomycin-resistant *E. faecalis*, are susceptible to ampicillin. In patients with IAIs, *E. faecium* is increasingly encountered, particularly in patients with hospital-acquired IAIs. In contrast to *E. faecalis*, nearly all strains of *E. faecium* are resistant to ampicillin and the growing prevalence of vancomycin-resistant strains is an area of concern, although the main clinical problem seems strictly related to BSIs. Indeed, a recent meta-analysis showed a higher mortality for vancomycin-resistant *E. faecium* BSIs compared with vancomycin-sensitive *E. faecium* BSIs (RR = 1.46; 95% CI 1.17–1.82) [195]. For patients with VanA-type vancomycin-resistant *E. faecium*, linezolid or daptomycin are the preferred agents. Both linezolid and daptomycin have good in vitro activity against vancomycin-resistant *E. faecium* although higher daily doses are needed [196]. For VanB-type resistant strains teicoplanin should be considered the preferred drug [197].

*What are the optimal daily doses and modality of administration of antibiotics in patients with IAIs?*

**19. In patients with IAIs and sepsis or septic shock, appropriate dose and administration mode of antibiotics should include: 1) proper loading dose, 2) extended or continuous infusion for beta-lactams and 3) knowledge of the peritoneal/biliary penetration rate (Low quality of evidence, strong recommendation).**

**20. Beta-lactam antibiotics exhibit good penetration rate in the peritoneal exudate fluid. In critically ill patients, continuous infusion should be implemented to grant optimal PK/PD target attainment at the infection site (Low quality of evidence, strong recommendation).**

Administering adequate doses of antibiotics should be based on the intrinsic pharmacokinetic (PK) and pharmacodynamic (PD) characteristics of each antibiotic class, the specific agent, and the specific pathophysiologic characteristics of the patient.

Antibiotic PD refers to the relationship existing between drug exposure and its capability to inhibit bacterial growth. The minimal inhibitory concentration (MIC) is the primary parameter used in vitro to assess the effectiveness of an antibiotic against its target bacteria. To obtain a therapeutic effect, in case of time-dependent antibiotics, namely the beta-lactams, the concentration at the infection site should exceed the MIC against the target bacteria for at least 40% of the dosing interval, and ideally for longer up to 400% (e.g. 100% fT > 4 × MIC); in case of concentration-dependent antibiotics, namely the aminoglycosides maximum concentration should be 8–tenfold higher than the MIC (C_max_/MIC ratio > 8–10) [198]. Antibiotic PK describes how antibiotics are absorbed, distributed, metabolized, and eliminated from the body, determining the time course and concentration of antibiotics in serum and tissues and at the site of infection. Suboptimal concentrations at the target site may have important clinical consequences such as therapeutic failure and promotion of AMR development, especially when clinical isolates have borderline in vitro susceptibility [198]. Tissue distribution is an important feature because high concentrations at the infection site may prevent resistance development. Generally, tissue distribution is higher for lipophilic agents compared to hydrophilic ones, but disease-related factors may concur with differential tissue distribution [199]. In patients with severe IAIs, increased doses may be needed to attain adequate concentrations of ceftazidime, meropenem, and imipenem [200–202]. The findings of an observational prospective study including critically ill patients with suspicion of IAI needing surgery and empirical therapy with a beta-lactam were published in 2020 [203]. It was found that high doses of beta-lactams were able to attain 100% serum fT > 4 × MIC within the first 24 h in as much as 78% of critically ill patients having severe IAIs. To define optimal beta-lactam dosing, the PK/PD target should consider both tissue penetration rate and local antimicrobial susceptibility.

When treating abdominal sepsis, clinicians must be aware that drug pharmacokinetics may significantly vary between patients due to the changeable pathophysiology of sepsis and must also consider the pathophysiological and immunological status of the patient [204].

The “dilution effect”, also called as “third spacing” phenomenon, must be considered when administering the loading dose of hydrophilic antibacterial agents such as beta-lactams, aminoglycosides, and glycopeptides, which distribution is limited to the extracellular space. Otherwise, the dilution effect may cause underexposure at the infection site, namely in the peritoneal fluid, and may cause treatment failure and/or resistance development.

Generally, to ensure prompt achievement of adequate therapeutically effective drug exposure at the infection site in patients with sepsis or with septic shock the loading dose of beta-lactams or glycopeptides should be 1.5-fold higher than the standard one used in clinically stable patients [204]. Afterwards, the maintenance doses should be based on the degree of the patient’s renal function and should be reassessed daily, because the changeable pathophysiological conditions of the critically ill patients may significantly affect drug disposition. Maintenance doses of renally excreted drugs must be decreased in patients with impaired renal function and must be increased in patients with augmented renal clearance (a creatinine clearance > 130 mL/min) [204, 205]. Serum creatinine is an unreliable marker of renal function in critically ill patients. Urinary creatinine clearance should be measured to properly assess the renal function [206].

Dosing regimens should depend on the time-dependency or concentration-dependency antibacterial activity of the selected agent. Beta-lactams exhibit time-dependent activity which is optimal when trough concentrations (C_min_) persist above the MIC, namely C_min_ > MIC [204]. Intensified frequency dosing, prolonged infusions and/or continuous infusions may improve the likelihood of achieving this target [204]. Under the same daily dose, prolonged or continuous infusions may maximize the attainment of C_min_ > MIC. Large randomized controlled trials comparing continuous *versus* intermittent infusion of piperacillin/tazobactam in patients with cIAIs [204] as well as of piperacillin/tazobactam, ticarcillin/clavulanate or meropenem in patients with severe sepsis [204], did not find improvement in clinical outcomes. However, the generalizability of these findings should not be extended to patients having high severity of illness and/or infections caused by borderline susceptible pathogens with high MIC, for whom the clinically relevant benefit predicted by the PK/PD theory should be the greatest. This has been supported by some retrospective studies [207–209]. Consequently, prolonged, or continuous infusions of beta-lactam agents should be considered beneficial for treating severely critically ill patients with abdominal sepsis, especially in settings with a high prevalence of MDR pathogens.

A recent multicentre randomized trial [210] showed that, in critically ill patients with sepsis, continuous infusion of meropenem was not superior to intermittent infusion in reducing mortality and emergence of pandrug-resistant or extensively drug-resistant bacteria at day 28. However, it has been argued that some bias might have affected the findings and limited the possibility of drawing definitive conclusions, namely a relatively long duration of hospitalization before randomization, the baseline severity of the underlying condition and the relatively small sample size [211]. Overall, prolonged or continuous infusions of beta-lactam agents should therefore be considered as an added value when treating critically ill patients with abdominal sepsis. Conversely, antibiotics with concentration-dependent activity may maximize their effect when attaining a peak plasma concentration (C_max_) to MIC (C_max_/MIC) ratio > 8–10, so once-daily pulse dosing should be the preferred method of administration [204].

Regarding aminoglycosides, once-daily dosing is beneficial also in terms of decreasing the nephrotoxicity risk compared to multiple daily dosing because accumulation in the renal cortex, related to carriers, may be saturated more effectively with the former administration mode [212].

**21. Antibiotics with good biliary excretion should be used for treating biliary tract infections even if supportive clinical studies are currently lacking (Very low quality, strong recommendation).**

**22. Data concerning PK/PD target attainment of novel beta-lactam antibiotics in biliary tract infections are currently unavailable (No recommendation).**

The bacterial species most often isolated in the biliary tract infections are the same isolated in IAIs and include Gram-negative aerobes, such as *E. coli* and *K. pneumoniae* and anaerobes, especially *B. fragilis* [213]. The efficacy of antibiotic treatment of biliary tract infections may depend on effective biliary concentrations, even if clinical data supporting this contention are currently lacking [214]. An interesting review of the pharmacokinetics of antibiotics penetrating the bile and the gallbladder wall was published in 2020 [214]. The efficacy and pharmacokinetics of 50 antibiotics were analysed, and overall, most of them exhibited a valuable biliary penetration translating into clinical efficacy. Only seven antibiotics (namely amoxicillin, cefadroxil, cefoxitin, ertapenem, gentamicin, amikacin, and trimethoprim/sulfamethoxazole) had poor biliary penetration rates. Three antibiotics (namely ceftibuten, ceftolozane/tazobactam, and doripenem) showed favourable clinical outcomes regardless of unavailability of pharmacokinetic studies assessing their biliary penetration rate. Conflicting efficacy was reported for ampicillin despite adequate biliary penetration, whereas conflicting pharmacokinetic data were reported with cefaclor and moxifloxacin. Even in the absence of supportive clinical studies, the authors concluded that antibiotics with good biliary penetration profiles may have a place in the treatment of biliary tract infections.

*What is the optimal antifungal treatment in patients with IAIs?*

**23. In patients with septic shock and multi-organ failure, empiric antifungal therapy for Candida species should be considered for patients with hospital-acquired cIAIs, especially those with recent abdominal surgery or proximal gastrointestinal anastomotic leak or for patients with community-acquired infections at high risk (Low quality of evidence, strong recommendation).**

The epidemiological role of *Candida* spp. in patients with IAIs has not yet been conclusively defined [215]. Empirical antifungal therapy for *Candida* spp. is typically not recommended for patients with CA-IAIs, with the exceptions of critically ill patients with septic shock, multiple organ failure and risk factors for *Candida* spp. infections or immunocompromised patients (due to neutropenia or concurrent administration of immunosuppressive agents, such as glucocorticosteroids, chemotherapeutic agents, and immunomodulators). In 2016, the IDSA guidelines for the treatment of invasive candidiasis were developed and addressed intra-abdominal candidiasis (IAC) [216]. IDSA guidelines suggest considering empiric antifungal therapy for patients with clinical evidence of IAIs and significant risk factors for candidiasis, including recent abdominal surgery, anastomotic leaks, or necrotizing pancreatitis, who are doing poorly despite treatment for bacterial infections. While patients with abdominal sepsis may not in general benefit from empiric antifungal agents, some patients with particular risk factors for fungal infection who fail to improve after some days of broad-spectrum antibiotic therapy are at increased risk of having invasive candidiasis. The most recent ESICM/ESCMID guidelines recommendations state that empiric antifungal treatment may be considered in patients with sepsis or septic shock at high risk for *Candida* spp. infections [217]. These recommendations are mainly based on the results of several observational studies, in which an early antifungal therapy was associated with a better outcome. However, no evidence favouring an early empirical antifungal therapy comes from prospective studies.

In a recent German trial aiming to evaluate the diagnostic value of Beta-D-glucan in invasive candidiasis compared with standard cultures, in both arms, empiric treatment was discouraged, confirming the increasing doubts about the role of extended empiric treatment against *Candida* spp. [218]. In any IAIs associated with sepsis or septic shock, extended empiric treatment, generating an excess of exposure to antifungal agents, is probably an important driver of non-*albicans*-resistant species selection [219]. There is a critical need for more robust clinical trials and surveillance of antifungal resistance to enhance patient care and optimise treatment outcomes. Such evidence will help to improve the existing guidelines and contribute to a more personalised and effective approach to treating this serious medical condition. A possible solution to the conundrum of empiric antifungal therapy in IAIs could be based on the application in the clinical practice of the high negative predictive value of 1,3-beta-d-Glucan, used when negative for an early withdrawal of antifungal agents [220].

**24. The use of echinocandins for the management of intrabdominal candidiasis may be affected by suboptimal PK/PD target attainment. In critically ill patients dose adjustments may be needed if echinocandins are used (Very low quality of evidence, strong recommendation).**

**23 In patients at high risk for intra-abdominal candidiasis, liposomal amphotericin B may be used as pre-emptive therapy waiting for the result of the 1,3-beta-D-Glucan (if 1,3-beta-d-Glucan test is available) (Very low quality of evidence, weak recommendation).**

Although traditionally recommended by guidelines, the role of echinocandins has been debated in recent years [221]. Indeed, there is mounting evidence in the literature showing that echinocandin exposure in the abdomen could be suboptimal in critically ill patients and that dosing increases guided by Therapeutic Drug Monitoring may be needed.

In 2021 Welte et al*.* assessed the pharmacokinetic behaviour and the antifungal activity of anidulafungin, micafungin, and caspofungin in ascites fluid and plasma of critically ill adults treated for suspected or proven invasive candidiasis [222]. The study demonstrated that standard daily doses of anidulafungin, micafungin, or caspofungin resulted in ascites fluid concentrations preventing relevant proliferation of *C. albicans* and *C. glabrata*, but did not warrant reliable eradication. Another recent study showed moderate penetration of echinocandins into the peritoneal fluid. These levels were below the threshold of resistance mutant selection published by other authors, justifying a potential risk of resistance in patients with prolonged treatment with echinocandins and suboptimal control of abdominal infection [223]. Controlled clinical studies on the treatment of IAC are currently lacking. Recent reports suggest dose adjustments in patients with reduced albumin levels, increased weight, and severe infections. Most studies report 20% lower exposures in critically ill patients. Therefore, concentration might be insufficient although no PK/PD target in the peritoneum has been defined for echinocandins [224].

While azoles are no longer considered the first choice in critically ill patients, due to the high level of resistance and/or the high risk of drug-drug interactions, a qualified alternative could be represented by Liposomal Amphotericin B. Liposomal amphotericin B is a lipid-based formulation of amphotericin B [225]. The liposomal formulation allows an increase in doses compared with the deoxycholate formulation. It has improved antifungal efficacy and an affordable lower risk of side effects and nephrotoxicity. The potential of drug interactions is negligible as well as the risk of resistance selection. It has concentration-dependent fungicidal activity, a prolonged half-life and showed an extended post-antifungal effect in time-kill studies [226]. Moreover, due to the lipophilic characteristics, it might be less affected by pathophysiological changes than hydrophilic drugs like echinocandins. Clinical data about the use of liposomal amphotericin B in the setting of IAIs are quite limited. However, its use may be rationale and it may be considered as a potential first-line option, also because its acceptable safety has been reported in the ICU setting [227]. Very recently a monocentric experience showed that a single 5 mg/kg pulse dose of liposomal amphotericin B as pre-emptive therapy in patients at high risk for IAC was safe and cost-effective while waiting for the result of the 1,3-beta-D-Glucan test [228].

Finally, the T2Candida magnetic resonance assay is a direct-from-blood pathogen detection assay that delivers a result within 3–5 h, targeting the most clinically relevant *Candida* species. Between February 2019 and March 2021, a study including consecutive patients admitted to an ICU or surgical high-dependency unit due to gastrointestinal surgery or necrotizing pancreatitis was conducted [229]. Blood samples were tested with both T2Candida and 1,3-β-D-glucan. Of 134 evaluable patients, 13 (10%) had proven IAC. Two of the 13 patients with IAC (15%) had concurrent candidemia. The sensitivity, specificity, positive predictive value, and negative predictive value for T2Candida were respectively, 46%, 97%, 61%, and 94%. The sensitivity, specificity, positive predictive value, and negative predictive value for 1,3-β-D-glucan were 85%, 83%, 36%, and 98%. The performance of T2Candida was similar to that of 1,3-β-D-glucan for candidemic IAC. However, the T2Candida showed lower sensitivity for non-candidemic IAC (36% *versus* 82%). In conclusion, T2Candida may be considered a complement to 1,3-β-D-glucan in the clinical management of high-risk surgical patients.

## Conclusions

An effective antimicrobial strategy for managing IAIs requires a correct balance between optimizing empiric therapy to improve clinical outcomes and curbing excessive antimicrobial use to mitigate the emergence of multidrug-resistant strains. Shared pathways for the most common IAIs are illustrated in appendices 1–9.

To prevent the selection and the spread of AMR and to treat infections correctly, we need Culture, Methods, Experience, Honesty, Organization and Multidisciplinary approach.

The following principles should be the basis of ethical in-hospital antimicrobial stewardship:

1. Choose antimicrobials using a risk assessment-based approach.
2. Do not be impulsive in starting antimicrobial therapy.
3. Use appropriate microbiology resources.
4. Avoid redundant prescriptions and useless combinations.
5. Be aware of PK/PD features.
6. Rethink early how antibiotics are prescribed.
7. Shorten therapy duration.
8. Define the right indications of therapy for new drugs.
9. Work together.

## Data Availability

Not applicable.

## Appendix 1: Management of acute appendicitis

### Diagnosis

#### Clinical signs and symptoms

- Abdominal pain: it usually has a gradual onset and increases with intensity over time. It is usually relieved in the supine position and aggravated by coughing or abdominal movements. Typically, there may be a short history of migration of the pain from the peri-umbilical region to the right low quadrant.
- Nausea and/or vomiting soon after abdominal pain begins.
- Fever.
- Tenderness localized in the right low quadrant (typically in complicated acute appendicitis).

#### Laboratory markers

- Increased white blood cell count.
- Leucocyte shift to left (> 75%).
- Increased C-reactive protein.

#### Severity scores

- Alvarado score.
- the Andersson appendicitis inflammatory response (AIR).
- Adult appendicitis score (AAS).

#### Imaging

- US.
- CT with IV contrast.

#### Imaging findings

- Diameter of the appendix > 6 mm.
- Single wall thickness ≥ 3 mm.
- Increased echogenicity of local mesenteric fat.
- Appendicolith: hyperechoic with posterior shadowing.
- Free fluid surrounding appendix.
- Local abscess formation.
- Enlarged local mesenteric lymph nodes.
- Thickening of the peritoneum.

### Treatment

#### Uncomplicated appendicitis

- Laparoscopic appendectomy (current standard surgical treatment) or open appendectomy. One shot prophylaxis if early intervention. No post-operative antibiotics.
- Conservative treatment. Antibiotic therapy without surgery. It is less effective in the long-term because of significant recurrence rates, requires a CT proven diagnosis of uncomplicated appendicitis, and there may be a risk of perforation increasing with preoperative delay.

#### Complicated appendicitis

- Percutaneous drainage as bridge to intervention (if periappendicular absess) in immunocompent and no critically ill patients.
  +
- Antibiotic therapy until intervention and for 2–4 subsequent days.
- Laparoscopic or open appendectomy as an alternative.
  +
- Antibiotic therapy for 2–4 days in immunocompetent and no critically ill patients if source control is adequate.
- Antibiotic therapy up to 7 days based on clinical conditions and inflammation indices if source control is adequate in immunocompromised or critically ill patients.

Patients who have ongoing signs of infection or systemic illness beyond 7 days of antibiotic treatment warrant a diagnostic investigation.

### Empiric antibiotic therapy

**Complicated appendicitis**

**Normal Renal Function**

**Adequate source control**

**Immunocompetent and no critically ill patients**

Amoxicillin/Clavulanate 2 g/0.2 g q8h.

*In patients with documented beta-lactam allergy:*

Eravacycline 1 mg/kg q12h.

or

Tigecycline 100 mg LD then 50 mg q12h.

**Critically ill or Immunocompromised patients**

**Adequate source control**

Piperacillin/tazobactam 6 g/0.75 g LD then 4 g/0.5 g q6h or 16 g/2 g by continuous infusion.

*In patients with documented beta-lactam allergy:*

Eravacycline 1 mg/kg q12h.

**Patients with inadequate/delayed source control or at high risk of infection with community-acquired ESBLs-producing Enterobacterales**

Ertapenem 1g q24h.

or

Eravacycline 1 mg/kg q12h.

**If septic shock**

One of the following antibiotics

Meropenem 1 g q6 h by extended infusion or continuous infusion

or

Doripenem 500 mg q8h by extended infusion or continuous infusion

or

Imipenem/cilastatin 500 mg q6h by extended infusion

or

Eravacycline 1 mg/kg q12h.

## Appendix 2: Management of acute cholecystitis

### Diagnosis

#### Clinical signs and symptoms

- Abdominal pain in the right upper quadrant of the abdomen.
- Murphy’s sign.
- Fever.
- Abdominal tenderness, palpable gallbladder lump (sign of complicated acute cholecystitis).

#### Laboratory markers

- Increased white blood cell count.
- Leucocyte shift to left (> 75%).
- C-reactive protein.

#### Imaging

- US (investigation of choice in patients with suspected acute cholecystitis).
- CT with IV contrast.
- Magnetic resonance cholangiopancreaticography (MRCP) (in patients with suspected common bile duct stones).

#### Imaging findings

- Pericholecystic fluid (fluid around the gallbladder).
- Distended gallbladder, edematous gallbladder wall.
- Gallstones (impacted in cystic duct).
- Murphy’s sign can be elicited on ultrasound examination.

### Treatment

#### Uncomplicated cholecystitis

- EARLY TREATMENT: Early (within 7–10 days the onset of symptoms) laparoscopic/open cholecystectomy. One shot prophylaxis if early intervention. **No** post-operative antibiotics.
- DELAYED TREATMENT: Antibiotic therapy and planned delayed cholecystectomy (second option) (not in immunocompromised patients). Antibiotic therapy for no more than 7 days.

#### Complicated cholecystitis

Laparoscopic cholecystectomy with open cholecystectomy as an alternative

+

Antibiotic therapy for 4 days in immunocompetent and no critically ill patients if source control is adequate.

Antibiotic therapy up to 7 days based on clinical conditions and inflammation indices if source control is adequate in immunocompromised or critically ill patients.

Patients who have ongoing signs of infection or systemic illness beyond 7 days of antibiotic treatment warrant a diagnostic investigation.

Cholecystostomy may be an option for acute cholecystitis in patients with multiple comorbidities and unfit for surgery patients who do not show clinical improvement after antibiotic therapy for days. Antibiotic therapy for 4 days. Cholecystostomy is inferior to cholecystectomy in terms of major complications for critically ill patients. It should not be performed in critically ill or immunocompromised patients.

### Empiric antibiotic therapy

**Normal renal function**

**No critically ill and Immunocompetent patients**

**Adequate source control**

Amoxicillin/Clavulanate 2g/0.2g q8h.

*In patients with documented beta-lactam allergy:*

Eravacycline 1 mg/kg q12h

or

Tigecycline 100 mg LD then 50 mg q12h

**Critically ill or Immunocompromised patients**

**Adequate source control**

Piperacillin/tazobactam 6 g/75 g LD then 4 g/0.5 g q6h or 16 g/2 g by continuous infusion.

*In patients with documented beta-lactam allergy:*

Eravacycline 1 mg/kg q12h.

**Patients with inadequate/delayed source control or at high risk of infection with community-acquired ESBLs-producing Enterobacterales**

Ertapenem 1 g q24h

or

Eravacycline 1 mg/kg q12h

**If septic shock**

One of the following antibiotics:

Meropenem 1 g q6h by extended infusion or continuous infusion

or

Doripenem 500 mg q8h by extended infusion or continuous infusion

or

Imipenem/cilastatin 500 mg q6h by extended infusion

or

Eravacycline 1 mg/kg q12h.

## Appendix 3: Management of acute cholangitis

### Diagnosis

#### Clinical signs and symptoms

- Intermittent fever with rigors.
- Jaundice.
- Right upper quadrant abdominal pain.

#### Laboratory markers

- Increased white blood cell count.
- C-reactive protein.
- Procalcitonin.
- Total bilirubin.
- Direct bilirubin.
- Alkaline phosphatase.
- Gamma-glutamyl transpeptidase.
- Aspartate aminotransferase.
- Alanine aminotransferase.

#### Imaging

- US.
- CT with IV contrast.
- MRI.
- Endoscopic ultrasound (EUS).
- ERCP.

#### Imaging findings

- Dilatation of intra- and extra-hepatic bile ducts.
- Thickening of the bile duct wall.
- Intraluminal stones or sludge.

### Treatment

#### Biliary drainage

+

Antibiotic therapy for 4 days in immunocompetent and no critically ill patients if source control is adequate.

Antibiotic therapy up to 7 days based on clinical conditions and inflammation indices if source control is adequate in immuocompromised or critically ill patients.

Patients who have ongoing signs of infection or systemic illness beyond 7 days of antibiotic treatment warrant a diagnostic investigation.

The type and timing of biliary drainage should be based on the severity of the clinical presentation, and the availability and feasibility of drainage techniques, such as endoscopic retrograde cholangiopancreatography (ERCP), percutaneous transhepatic cholangiography (PTC), and open surgical drainage.

### Empiric antibiotic therapy

**Normal renal function**

**No critically ill and immunocompetent patients**

**Adequate source control**

Amoxicillin/Clavulanate 2 g/0.2 g q8h.

*In patients with documented beta-lactam allergy:*

Eravacycline 1 mg/kg q12h

or

Tigecycline 100 mg LD then 50 mg q12h.

**Critically ill or Immunocompromised patients**

**Adequate source control**

Piperacillin/tazobactam 6 g/75 g LD then 4 g/0.5 g q6h or 16 g/2 g by continuous infusion.

or

Eravacycline 1 mg/kg q12h.

**Patients with inadequate/delayed source control or at high risk of infection with community-acquired ESBLs-producing Enterobacterales**

Ertapenem 1 g q24h

or

Eravacycline 1 mg/kg q12h.

**If septic shock**

One of the following antibiotics:

Meropenem 1 g q6h by extended infusion or continuous infusion

or

Doripenem 500 mg q8h by extended infusion or continuous infusion

or

Imipenem/cilastatin 500 mg q6h by extended infusion

or

Eravacycline 1 mg/kg q12h.

## Appendix 4: Management of acute diverticulitis

### Diagnosis

#### Clinical signs and symptoms

- Abdominal pain in the left lower quadrant of the abdomen without vomiting.
- Elevated temperature.
- Tenderness localized in the left lower quadrant.

#### Laboratory markers

- Increased white blood cell count.
- Leucocyte shift to left (> 75%).
- C-reactive protein.
- Procalcitonin.

#### Imaging

- US.
- CT with IV contrast.

#### Imaging findings

- Intestinal wall thickening.
- Signs of inflammation in the pericolonic fat and thickening of the lateroconal fascia.
- Signs of intestinal perforation (extraluminal gas, intra-abdominal fluid).
- Pericolonic or distant abscess.

#### Uncomplicated diverticulitis

- Conservative treatment without antibiotics in patients with CT diagnosis of uncomplicated acute diverticulitis.
- Antibiotic therapy for no more than 7 days in patients with CT diagnosis of uncomplicated acute diverticulitis in immunocompromised/aged patients

#### Abdominal abscess

- Antibiotic therapy alone for 7 days in patients with small diverticular abscesses.
- Percutaneous drainage combined with antibiotic therapy for 4 days in large diverticular abscesses.

If percutaneous drainage of the abscess is not feasible or not available, in no critically ill patients and immunocompetent patients’ antibiotics alone could be considered the primary treatment.

If percutaneous drainage of the abscess is not feasible or not available, in critically ill patients and immunocompromised patients’ surgical intervention could be considered the primary treatment.

#### Diffuse peritonitis

- Primary resection and anastomosis with or without a diverting stoma (in clinically stable patients with no co-morbidities).
- Hartmann’s procedure (in critically ill patients and/or in patients with multiple major comorbidities).
- Laparoscopic peritoneal lavage and drainage suitable only for patients with purulent (but not fecal) peritonitis due to complicated diverticulitis. Very controversial.

+

Antibiotic therapy for 4 days in immunocompetent and no critically ill patients if source control is adequate.

Antibiotic therapy up to 7 days based on clinical conditions and inflammation indices if source control is adequate in immuocompromised or critically ill patients.

Patients who have ongoing signs of infection or systemic illness beyond 7 days of antibiotic treatment warrant a diagnostic investigation.

### Empiric antibiotic therapy

**Normal Renal Function**

**No critically ill and Immunocompetent patients**

**Adequate source control**

Amoxicillin/Clavulanate 2 g/0.2 g q8h.

*In patients with documented beta-lactam allergy:*

Eravacycline 1 mg/kg q12h

or

Tigecycline 100 mg LD then 50 mg q12h.

**Critically ill or Immunocompromised patients**

**Adequate source control**

Piperacillin/tazobactam 6 g/0.75 g LD then 4 g/0.5 g q6h or 16 g/2 g by continuous infusion.

or

Eravacycline 1 mg/kg q12h.

**Patients with inadequate/delayed source control or at high risk of infection with community-acquired ESBLs-producing Enterobacterales**

Ertapenem 1 g q24h.

or

Eravacycline 1 mg/kg q12h.

**If septic shock**

One of the following antibiotics:

Meropenem 1 g q6h by extended infusion or continuous infusion

or

Doripenem 500 mg q8h by extended infusion or continuous infusion

or

Imipenem/cilastatin 500 mg q6h by extended infusion

or

Eravacycline 1 mg/kg q12h.

## Appendix 5: Management of non-traumatic small bowel perforation

### Diagnosis

#### Clinical signs and symptoms

- Severe, sudden-onset periumbilical pain, which can become generalized.
- Abdominal tenderness.
- Fever.

#### Laboratory markers

- Increased white blood cell.
- Leucocyte shift to left (> 75%).
- C-reactive protein.

#### Imaging

- US.
- CT with IV contrast.
- CT angio (if there is suspicion of acute mesenteric ischemia).

### Treatment

- Open or laparoscopic small bowel segmental resection and primary anastomosis.
- In the setting of perforation due to small bowel ischemia, resection and delayed anastomoses at a second look are usually needed. Also, open or endovascular mesenteric vessel reconstruction may be needed.
- Open or laparoscopic resection and stoma creation or exteriorization of the perforation as a stoma (critically ill patients or severe inflammation and edema of the bowel, resulting in friable tissue which precludes anastomosis).

+

Antibiotic therapy for 4 days in immunocompetent and no critically ill patients if source control is adequate.

Antibiotic therapy up to 7 days based on clinical conditions and inflammation indices if source control is adequate in immuocompromised or critically ill patients.

Patients who have ongoing signs of infection or systemic illness beyond 7 days of antibiotic treatment warrant a diagnostic investigation.

### Antibiotic therapy

**Empiric Antibiotic Regimens; Normal Renal Function**

**Adequate source control**

Piperacillin/tazobactam 6 g/0.75 g LD then 4 g/0.5 g q6h or 16 g/2 g by continuous infusion.

*In patients with documented beta-lactam allergy:*

Eravacycline 1 mg/kg q12h

Or

Tigecycline 100 mg LD then 50 mg q12h.

**Patients with inadequate/delayed source control or at high risk of infection with community-acquired ESBLs-producing Enterobacterales**

Ertapenem 1 g q24h

or

Eravacycline 1 mg/kg q12h.

**If septic shock**

One of the following antibiotics:

Meropenem 1 g q6h by extended infusion or continuous infusion

or

Doripenem 500 mg q8h by extended infusion or continuous infusion

or

Imipenem/cilastatin 500 mg q6h by extended infusion

or

Eravacycline 1 mg/kg q12h.

## Appendix 6: Gastroduodenal ulcer perforation

### Diagnosis

#### Clinical signs and symptoms

- Severe, sudden-onset epigastric pain, which can become generalized.
- Abdominal tenderness.
- Fever.
- Abdominal distension, tenderness, and rigidity with masked liver dullness and absent bowel sounds.

#### Laboratory markers

- White blood cell count.
- Leucocyte left shift (> 75%).
- C-reactive protein.

#### Imaging

- US.
- CT with IV contrast.

#### Imaging findings

Signs of gastrointestinal perforation (extraluminal gas, intra-abdominal fluid).

Air pockets around the stomach and duodenum and thick reactive intestinal wall.

### Treatment

- Conservative treatment without surgery only in patients not eligible for surgical repair because of severe comorbidities.
- Laparoscopic/open simple or double-layer suture with or without an omental patch is a safe and effective procedure to address small perforated ulcers (standard procedure).
- Distal gastrectomy (large perforations near the pylorus; suspicion of malignancy).

+

Antibiotic therapy for 4 days in immunocompetent and no critically ill patients if source control is adequate.

Antibiotic therapy up to 7 days based on clinical conditions and inflammation indices if source control is adequate in immuocompromised or critically ill patients.

Patients who have ongoing signs of infection or systemic illness beyond 7 days of antibiotic treatment warrant a diagnostic investigation.

### Empiric antibiotic therapy

**Empiric Antibiotic Regimens; Normal Renal Function**

**No critically ill and Immunocompetent patients**

**Adequate source control**

Amoxicillin/Clavulanate 2 g/0.2 g q8h.

*In patients with documented beta-lactam allergy:*

Eravacycline 1 mg/kg q12h

or

Tigecycline 100 mg LD then 50 mg q12h.

**Critically ill or Immunocompromised patients**

**Adequate source control**

Piperacillin/tazobactam 6 g/0.75 g LD then 4 g/0.5 g q6h or 16 g/2 g by continuous infusion.

*In patients with documented beta-lactam allergy:*

Eravacycline 1 mg/kg q12h.

**Patients with inadequate/delayed source control or at high risk of infection with community-acquired ESBLs-producing Enterobacterales**

Ertapenem 1 g q24h

or

Eravacycline 1 mg/kg q12h.

**If septic shock**

One of the following antibiotics:

Meropenem 1 g q6h by extended infusion or continuous infusion

or

Doripenem 500 mg q8h by extended infusion or continuous infusion

or

Imipenem/cilastatin 500 mg q6h by extended infusion

or

Eravacycline 1 mg/kg q12h.

## Appendix 7: Post-operative peritonitis

### Diagnosis

#### Clinical signs and symptoms

- Fever.
- Abdominal pain.
- Abdominal tenderness.

#### Laboratory markers

- Increased white blood cell count.
- C-reactive protein.
- PCT.

#### Imaging

CT with IV contrast.

#### Imaging findings

- Signs of intestinal perforation such as extraluminal air bubbles, intra-abdominal fluid.
- Post-operative abscess.

### Treatment

**Localized abscess**

Percutaneous drainage.

+

Antibiotic therapy for 4 days in immunocompetent and no critically ill patients if source control is adequate.

Antibiotic therapy up to 7 days based on clinical conditions and inflammation indices if source control is adequate in immunocompromised patients.

Patients who have ongoing signs of infection or systemic illness beyond 7 days of antibiotic treatment warrant a diagnostic investigation and a multidisciplinary re-evaluation.

**Diffuse peritonitis**

Early surgical source control and maximal/broad spectrum antibiotic therapy. The inability to control the septic source is associated with an intolerably high mortality rate.

+

Antibiotic therapy to 7 days based on clinical conditions and inflammation indices if source control is adequate in immunocompromised or critically ill patients.

Patients who have ongoing signs of infection or systemic illness beyond 7 days of antibiotic treatment warrant a diagnostic investigation.

### Empiric antibiotic therapy

**Empiric Antibiotic Regimens; Normal Renal Function**

**Patients without gut colonization by MDR and Immunocompetent patients**

One of the following antibiotics:

Meropenem 1 g q6h by extended infusion or continuous infusion

or

Doripenem 500 mg q8h by extended infusion or continuous infusion

or

Imipenem/cilastatin 500 mg q6h by extended infusion

or

Eravacycline 1 mg/kg q12h.

**Patients with suspected MDR etiology based on epidemiological data and or gut colonization data and or specific risk factors**

Imipenem/cilastatin-relebactam 1.25 g q6h by extended infusion

or

one of the following antibiotics:

Meropenem/vaborbactam 2 g/2 g q8h by extended infusion or continuous infusion

Ceftazidime/avibactam 2.5 g q8h by extended infusion or continuous infusion + Metronidazole 500 mgq8h

+

one of the following antibiotics:

Linezolid 600 q 12 h

or

Teicoplanin 12 mg/kg every 12 h 3 LDs then 6 mg/kg q12 h.

*In patients with documented beta-lactam allergy:*

Eravacycline 1 mg/kg q12h.

+

*in patients at high risk for intra-abdominal candidiasis:*

Liposomal amphotericin B 5 mg/kg pulse dose as pre-emptive therapy waiting for the result of the 1,3-beta-D-Glucan test (if 1,3 beta-D-glucan test is available)

or

one of the following echinocandins (considering PK/PD principles):

Caspofungin 70 mg LD, then 50 mg q24h

Anidulafungin 200 mg LD, then 100 q24h

Micafungin 100 mg q24h.

## Appendix 8: Acute pancreatitis

### Diagnosis

#### Clinical signs and symptoms

- Fever.
- Abdominal pain.

#### Laboratory markers

- Lipase.
- Amylase.
- Increased white blood cell count.
- C-reactive protein.
- PCT (is the most sensitive laboratory test for detection of pancreatic infection, and low serum values appear to be strong negative predictors of infected necrosis).

#### Imaging

- US.
- CT with IV contrast.
- MRI.
- Endoscopic ultrasound (EUS).

### Treatment

Mild acute pancreatitis:

- General (regular) diet and advance as tolerated.
- Pain control with oral medications.
- Routine vital signs monitoring.

Moderately severe acute pancreatitis:

- Enteral Nutrition (oral, NG or NJ) If not tolerated, it is possible to use parenteral nutrition.
- IV pain medications.
- IV fluids to maintain hydration.
- Monitoring hematocrit, blood urea nitrogen, creatinine.
- Continuous vital signs monitoring.

Severe acute pancreatitis:

- Enteral Nutrition (oral, NG or NJ). If not tolerated, it is possible to use parenteral nutrition.
- IV pain medications.
- Early fluid resuscitation.
- Mechanical ventilation.

No specific pharmacological treatment except for organ support and nutrition should be given.

Prophylactic antibiotics in patients with acute pancreatitis are not associated with a significant decrease in mortality or morbidity. Thus, routine prophylactic antibiotics are no longer recommended for all patients with acute pancreatitis.

Antibiotics are always recommended to treat infected severe acute pancreatitis. However, the diagnosis is challenging due to the clinical picture that cannot be distinguished from other infectious complications or from the inflammatory status caused by acute pancreatitis.

- PCT.
- A CT- or EUS-guided fine-needle aspiration (FNA) for Gram stain and culture.
- Endoscopic retrograde cholangiopancreatography (ERCP) in patients with acute biliary pancreatitis and common bile duct obstruction should be performed as soon as possible.
- Laparoscopic cholecystectomy should be considered carefully in patients with moderately severe and severe acute biliary pancreatitis, as it may be associated with increased postoperative mortality and morbidity
- Clinical deterioration with signs or strong suspicion of infected necrotizing pancreatitis is an indication to perform intervention.
- When a patient deteriorates a step-up approach starting with percutaneous or endoscopic drainage may be indicated.
- The following are indications for surgical intervention:
- as a continuum in a step-up approach after percutaneous/endoscopic procedure with the same indications;
- abdominal compartment syndrome;
- acute on-going bleeding when endovascular approach is unsuccessful;
- bowel ischaemia or acute necrotizing cholecystitis during acute pancreatitis;
- bowel fistula extending into a peripancreatic collection.
- Postponing surgical interventions for more than 4 weeks after the onset of the disease results in less mortality
- In patients with severe acute pancreatitis unresponsive to conservative management of IAH/ACS, surgical decompression and use of open abdomen are effective in treating the abdominal compartment syndrome. However, the open abdomen should be avoided if other strategies can be used to mitigate or treat severe IAH in patients with acute pancreatitis.

### Antibiotic therapy

**Routine prophylactic antibiotics should be not prescribed for patients with acute pancreatitis.**

**Antibiotic therapy should be administered only to treat infected acute pancreatitis.**

**Empiric Antibiotic Regimens; Normal Renal Function**

**Patients without gut colonization by MDR and Immunocompetent patients**

One of the following antibiotics:

Meropenem 1 g q6h by extended infusion or continuous infusion

or

Doripenem 500 mg q8h by extended infusion or continuous infusion

or

Imipenem/cilastatin 500 mg q6h by extended infusion or continuous infusion

or

Eravacycline 1 mg/kg q12h.

**Patients with suspected MDR etiology based on epidemiological data and or gut colonization data and or specific risk factors**

Imipenem/cilastatin-relebactam 1.25 g q6h by extended infusion

or

one of the following antibiotics:

Meropenem/vaborbactam 2 g/2 g q8h by extended infusion or continuous infusion

Ceftazidime/avibactam 2.5 g q8h by extended infusion or continuous infusion + Metronidazole 500 mgq8h

+

one of the following antibiotics:

Linezolid 600 q 12 h

Teicoplanin 12 mg/kg every 12 h 3 LDs then 6 mg/kg q12 h

*In patients with documented beta-lactam allergy:*

Eravacycline 1 mg/kg q12h.

+

*in patients at high risk for intra-abdominal candidiasis:*

Liposomal amphotericin B 5 mg/kg pulse dose as pre-emptive therapy waiting for the result of the 1,3-beta-D-Glucan test (if 1,3 beta-D-glucan test is available)

or

one of the following echinocandins (considering PK/PD principles):

Caspofungin 70 mg LD, then 50 mg q24h

Anidulafungin 200 mg LD, then 100 q24h

Micafungin 100 mg q24h

## Abbreviations

ACS: Abdominal Compartment Syndrome
AGNB: Aerobic Gram-Negative Bacteria
AMR: Antimicrobial Resistance
BSIs: Bloodstream Infections
CA-IAIs: Community-Acquired Intra-Abdominal Infections
CAZ-AVI: Ceftazidime-Avibactam
CA-UTIs: Catheter-Associated Urinary Tract Infections
CLA-BSIs: Central Line-Associated Bloodstream Infections
CI: Confidence Interval
cIAIs: Complicated Intra-abdominal Infections
CPE: Carbapenemase-Producing *Enterobacterales*
CRP: C-Reactive Protein
CT: Computed Tomography
ECDC: European Centre for Disease Prevention and Control
ERCP: Endoscopic Retrograde Cholangiopancreatography
ESBLs: Extended-Spectrum Beta-Lactamases
ESCMID: European Society of Clinical Microbiology and Infectious Diseases
EUS: Endoscopic Ultrasound
HAIs: Hospital-Acquired Infections
HAP: Hospital-Acquired Pneumonia
IAC: Intra-Abdominal Candidiasis
IAH: Intra-Abdominal Hypertension
IAIs: Intra-Abdominal Infections
IAP: Intra-Abdominal Pressure
ICU: Intensive Care Unit
IDSA: Infectious Disease Society of America
IMP: Active-on-Imipenem beta-lactamase
IQR: Interquartile Range
IV: Intravenous
KPC: *K. pneumoniae* Carbapenemase
KPC-Kp: *K. pneumoniae* Carbapenemase-producing *K. pneumoniae*
MAP: Mean Arterial Pressure
MBLs: Metallo-Beta-Lactamases
MDR: Multidrug-Resistant
MIC: Minimal Inhibitory Concentration
MRI: Magnetic Resonance Imaging
NDM: New Delhi Metallo-beta-lactamase
OR: Odds Ratio
PCT: Procalcitonin
PD: Pharmacodynamics
PK: Pharmacokinetics
RCTs: Randomized controlled trials
SSIs: Surgical Site Infections
SVR: Systemic Vascular Resistance
US: Ultrasonography
VAP: Ventilator-Associated Pneumonia
VIM: Verona Integron-encoded Metallo beta-lactamase
WSES: World Society of Emergency Surgery
